# Notch2 pathway mediates breast cancer cellular dormancy and mobilisation in bone and contributes to haematopoietic stem cell mimicry

**DOI:** 10.1038/s41416-019-0501-y

**Published:** 2019-06-26

**Authors:** Mattia Capulli, Dayana Hristova, Zoé Valbret, Kashmala Carys, Ronak Arjan, Antonio Maurizi, Francesco Masedu, Alfredo Cappariello, Nadia Rucci, Anna Teti

**Affiliations:** Department of Biotechnological and Applied Clinical Sciences, University of L’Aquila Via Vetoio – Coppito 2, 67100 L’Aquila, Italy

**Keywords:** Cancer microenvironment, Breast cancer, Bone metastases, Cancer microenvironment, Breast cancer

## Abstract

**Background:**

Recurrence after >5-year disease-free survival affects one-fifth of breast cancer patients and is the clinical manifestation of cancer cell reactivation after persistent dormancy.

**Methods:**

We investigated cellular dormancy in vitro and in vivo using breast cancer cell lines and cell and molecular biology techniques.

**Results:**

We demonstrated cellular dormancy in breast cancer bone metastasis, associated with haematopoietic stem cell (HSC) mimicry, in vivo competition for HSC engraftment and non-random distribution of dormant cells at the endosteal niche. Notch2 signal implication was demonstrated by immunophenotyping the endosteal niche-associated cancer cells and upon co-culture with sorted endosteal niche cells, which inhibited breast cancer cell proliferation in a Notch2-dependent manner. Blocking this signal by in vivo acute administration of the γ-secretase inhibitor, dibenzazepine, induced dormant cell mobilisation from the endosteal niche and colonisation of visceral organs. Sorted Notch2^HIGH^ breast cancer cells exhibited a unique stem phenotype similar to HSCs and in vitro tumour-initiating ability in mammosphere assay. Human samples confirmed the existence of a small Notch2^HIGH^ cell population in primary and bone metastatic breast cancers, with a survival advantage for Notch2^HIGH^ vs Notch2^LOW^ patients.

**Conclusions:**

Notch2 represents a key determinant of breast cancer cellular dormancy and mobilisation in the bone microenvironment.

## Background

Tumour recurrence after a long disease-free time unveils the existence of a subpopulation of tumour cells that becomes quiescent and undergoes dormancy. The concept of tumour dormancy implies a prolonged asymptomatic period in which the cancer cells cease to proliferate until a triggering stimulus causes them to regain their function.^1^ Given the high mortality rate of tumour relapse, cancer dormancy is of great clinical importance and needs thorough investigation.

In breast carcinomas, disseminated tumour cells are usually directed to one of the most common metastatic sites, the bone.^2,3^ Clinical evidence shows that approximately 20% of breast cancer patients who are apparently healed by successful therapies may relapse in the bone up to 15 years later.^4^ Moreover, according to previous reports from our group and others,^5–7^ in their metastatic process breast cancer cells may transit through the bone before spreading to other organs. The bone marrow is a very dynamic organ in which highly demanding processes, such as haematopoiesis and bone remodelling, take place. Consequently, inside the bone marrow cavity, neoplastic cells are nourished by a plethora of signals received by the osteogenic and the haematogenous microenvironments. In most cases, these signals stimulate tumour cell growth and enhance aggressiveness.^8^ Intriguingly, inside this dynamic organ, there are the long-term haematopoietic stem cells (LT-HSCs), characterised by multipotent stemness and prolonged quiescence, reminiscent of dormancy.

Under physiological conditions, LT-HSCs are located in specific areas of the bone marrow adjacent to the trabecular bone, interacting with the HSC niche.^9,10^ HSC niches have been identified in the endosteal region and in the perivascular/vascular region. According to some reports, LT-HSC quiescence in the endosteal niche is supported by a specific subpopulation of osteoblast-like cells lining the endosteal surface of the trabecular bone, named spindle-shaped N-cadherin^+^/CD45^−^ osteoblasts (SNOs).^11^ However, others ruled out the involvement of this osteoblast subpopulation in LT-HSC maintenance.^12^

SNO cells, which were first described in 2003,^9^ make up a small proportion of cells positioned at the endosteal surface. SNOs induce the quiescent status of LT-HSCs via cell-to-cell contact and paracrine signals, impeding their exhaustion.^13^ In a previous study, it was observed that bone marrow LT-HSCs drastically diminish in number with the depletion of osteogenic cells, confirming the importance of their co-existence.^14^ In line with these observations, we hypothesised that the cancer cells entering the bone and becoming dormant would be subjected to the same environmental cues as LT-HSCs, sharing similar stemness properties and niche interactions. Hypothetically, a subpopulation of cancer cells that develop the ability to exploit the LT-HSC niche to become quiescent could have a survival advantage, being less sensitive to the host immune response and/or to cytotoxic treatments that mostly impinge actively dividing cells. In support of this theory, conventional chemotherapy is not effective on dormant breast cancer cells.^15^ Furthermore, it has been observed that disseminated cancer cells can mimic the communication of HSCs with the surrounding bone marrow stem cell habitat using various pathways, including Jagged1, thus maintaining their non-proliferative status^16,17^ until signals trigger their mobilisation.

A crucial role in the communication of LT-HSCs with SNOs is played by the Jagged1 partner, Notch. In mammals, four types of Notch exist, Notch1–4, which all interact with ligands from the Delta and Jagged1 family.^18^ Intriguingly, Jagged1 expressed by endosteal cells has been shown to play a role in the bone colonisation by cancer cells.^19^ Most importantly, the Notch pathway accounts for the development of HSCs during embryogenesis,^20^ as well as for the stem cell maintenance of normal self-renewing or malignant stem cells.^21,22^

Based on this background information, we hypothesised that the mechanisms driving the cell cycle arrest of breast cancer cells near the endosteal niche are similar to the mechanisms inducing the non-proliferating status of LT-HSCs. We focussed on the Notch signalling pathway hypothesising that this is one of the main drives of breast cancer cellular dormancy, mobilisation and stemness in the bone marrow.

## Methods

### Animals

Procedures involving animals and their care were conducted in conformity with national and international laws and policies (European Economic Community Council Directive 86/609, OJ L 358, 1, December 12, 1987; Italian Legislative Decree 116/92, *Gazzetta Ufficiale della Repubblica Italiana* no. 40, February 18, 1992; National Institutes of Health Guide for the Care and Use of Laboratory Animals, National Institutes of Health Publication no. 85–23, 1985). The procedures were approved by the Institutional Ethical Review Board of the University of L’Aquila and by the Ministry of Health. The study was conducted according to the Animal Research Reporting In Vivo Experiments (ARRIVE) requirements (Supplementary Table 1).

### Human samples

Archive human primary breast cancers and bone metastases were employed for immunohistochemical studies. The procedures were approved by the Institutional Ethical Review Board of the University of L’Aquila.

### Primary osteoblast cell isolation

Murine osteoblasts were isolated from the calvarias of 7–10-day-old CD1 mice. Calvarias underwent 3 steps of incubation at 37 °C with a digestion solution containing trypsin (SAFC Biosciences, cat: 85450 C) (25 mg/ml) and clostridial collagenase (Sigma-Aldrich, cat: C8051) (1 mg/ml) in Hanks’ Balanced Salt Solution (EuroClone, cat: ECB4007L). Cells from the second and third digestions were osteoblast enriched.

### Breast cancer cell culture

Human breast cancer cell lines (MDA-MB-231, luciferase- or turboGFP-transfected MDA-MB-231 and MCF-7) and mouse breast cancer cell lines (4T1) were used for all experiments. The cells were maintained in high glucose Dulbecco’s Modified Eagle Medium (DMEM, EuroClone, cat: ECB7501L) with the addition of 1% glutamine and penicillin–streptomycin (Euroclone, cat: ECB3001D). The medium contained 10% foetal bovine serum (Life Technologies, cat: 26140-079) as provision of nutrients.

### Notch silencing

TurboGFP-positive breast cancer cells were transfected with small interfering RNAs (siRNAs) against human Notch1–4 (Dharmacon, smartpool, cat: L-007771-00-0005, L-012235-00-0005, L-011093-00-0005 and L-011883-00-0005) at concentrations of 25 (Notch1 and Notch3) or 50 nM (Notch2 and Notch4) or with scrambled (SCR) siRNA as control (Dharmacon, smartpool, cat: D-001810-10). Notch downregulation was evaluated by real-time reverse transcriptase–polymerase chain reaction (RT-PCR) after 48 h of silencing. Transfected cells were then detached without the use of proteolytic agents and seeded onto SNOs or NON-SNOs. After 1 h, unbound cancer cells were removed by extensive wash in phosphate-buffered saline (PBS) and bound cells were counted under an epifluorescence microscope. Counting was then repeated at 24, 48 and 72 h and results were expressed as fold change vs 1 h count.

### Vital cell labelling

MCF-7 or 4T1 cell suspensions and murine HSCs were incubated with the stable membrane interlinker, PKH67 (Sigma-Aldrich, cat: MIDI26 or MIDI67), fluorescing in red (*λ* 567 nm) or fluorescing in green (*λ* 488 nm) respectively, following the manufacturer’s instructions, or labelled with the CMFDA (CellTracker™ Green CMFDA Dye, ThermoFisher cat: C2925).

### RNA extraction and real-time RT-PCR

RNA was extracted using TRIzol® (Life Technologies, cat: 15596018) according to the manufacturer’s instructions. Quality control was performed by agarose gel electrophoresis. RNA was quantified by Nanodrop®, using an absorbance of 260 nm wavelength. RNA purity was assessed by evaluation of 260/280 nm wavelength ratio. Two μg of RNA was retro-transcribed into cDNA using a cDNA synthesis Kit (ThermoFisher cat: K1622). Real-time PCR was carried out using Sybr/Hi-Rox Sensimix (Bioline, cat: QT605-05) and primer pairs for the specific genes of interest (Supplementary Table 2), using the housekeeping gene *GAPDH* as a normalisation control.

### Protein extraction and Western blot

Western blot analysis was used to detect protein expression in breast cancer cells. Cells were lysed in standard RadioImmunoPrecipitation Assay (RIPA) buffer (1 M Tris/HCl, pH 7.4, 1 M NaCl, Nonidet P-40, 10% sodium deoxycholate, 0.5 M ethylene-diamine-tetra-acetic acid (EDTA), pH 8, 0.1 M NaF, 20 mM Na_3_VO_4_, dH_2_0, 0.1 M PMSF) containing 1% protease inhibitor cocktail (Sigma-Aldrich; cod: P8340) and 10 μM sodium fluoride. Protein concentration was quantified using the Bradford assay. Total protein lysate (50 μg) was resolved by sodium dodecyl sulfate-polyacrylamide gel electrophoresis (BioRAD, UK), immunoblotted with primary antibody for Notch2 (Santa Cruz Biotechnology cat: sc5545) or β-actin (Santa Cruz Biotechnology cat: sc47778) at a 1:200 dilution, overnight at 4 °C, detected by horseradish peroxidase (HRP)-conjugated secondary antibodies (all at 1:1000 dilution, Santa-Cruz Biotechnology) and enhanced chemiluminescence (Thermo Scientific, cat: 34080) on a ChemiDoc® imaging system (Bio-Rad).

### Flow cytometry

Cells were detached and suspended in sterile sorting buffer containing 5% bovine serum albumin (BSA) (Sigma-Aldrich, cat: A2153) and 0.5 M EDTA (Life Technologies, cat: 15576-028) in DPBS (EuroClone, cat: ECB4004L). Cell suspensions were incubated for 1 h at 4 °C with 10 μl per 10^7^ cells of primary antibodies against Notch2 (Santa Cruz Biotechnology, cat: sc5545; or Miltenyi Biotec, cat: 130-096-980), Notch1 (Santa Cruz Biotechnology, cat: sc32745), CD34 (Santa Cruz Biotechnology, cat: sc7324), c-Kit (Santa Cruz Biotechnology, cat: sc17802), CD24 (BD Pharmingen, cat: 555426) and CD44 (Santa Cruz Biotechnology, cat: sc6786), along with 3 μl per 10^7^ cells of a fluorescent secondary antibody [*λ* 488 nm chicken anti-mouse (Life Technologies A21200), *λ* 594 nm goat anti-rabbit (Invitrogen by ThermoFisher Scientific, A11012), *λ* 488 nm streptavidin conjugate (Life Technologies, S32231), *λ* 488 nm Goat anti-mouse (Invitrogen by ThermoFisher Scientific, A11001) and *λ* 594 nm rabbit anti-goat (Life Technologies, A11080)], respectively. Afterwards, cells were analysed using FACScantoII equipped with the FACSDiva software.

### Magnetic-associated cell sorting (MACS)

Cells were detached and suspended in sterile sorting buffer containing 5% BSA (Sigma-Aldrich, cat: A2153) and 0.5 M EDTA (Life Technologies, cat: 15576-028) in DPBS (EuroClone, cat: ECB4004L). Cell suspensions were incubated for 20 min at 4 °C with primary antibody (3 μl/10^6^–10^7^ cells) against the surface protein of interest or with N-cadherin-Phycoerythrin (PE) (Novus, cat: A5-070516-PE) or Notch2-biotin (Miltenyi Biotec, cat: 130-096-980). Then cells were incubated in the same conditions with anti-PE antibody (Miltenyi Biotec, cat: 130-048-801) or streptavidin, respectively, conjugated to magnetic microbeads (20 μl/10^7^ cells) (Miltenyi Biotec, cat: 130-048-102). Afterwards, cells were run through the magnetic column to obtain separate antigen-depleted and antigen-enriched cell populations (Miltenyi Biotec, cat: 130-042-401).

To obtain HSC-enriched population, bone marrow was isolated from forelimbs and hindlimbs of 2-month-old female mice. Cells underwent a double sorting by MACS for depletion of lineage-associated antigens and enrichment of stemness marker. The total population of cells was incubated with a cocktail of biotinylated antibodies against the antigens CD5, CD45R, CD11b, Gr-1, and Ter-119, in order to deplete mature haematogenous cells (Miltenyi Biotec, cat: 130-090-858). Afterwards, the lineage-negative subpopulation was incubated with anti-Sca-1 (Miltenyi Biotec, cat: 130 092 529) antibody and run through the column to obtain a Lin^−^/Sca-1^+^ HSC population.

### Primary and secondary mammosphere-formation assay

Single-cell suspensions of sorted Notch2^HIGH^ or Notch2^LOW^ MDA-MB-231 cells were seeded into non-adhesive Petri dishes at a density of 10^3^/ml in serum-free DMEM, supplemented with 1% N2, 1% B27 (Life Technologies, cat: 17502048, 17504044), 1% penicillin/streptomycin and 1% L-glutamine. Growth was permitted for 1 week in a humidified CO_2_ incubator (5% CO_2_, 37 °C). For secondary formation assays, primary mammospheres were trypsinised to obtain single-cell suspensions and plated under the same conditions. Counting and imaging was performed using the SXView Software.

### Alkaline phosphatase (ALP) staining

Osteoblasts were fixed with 4% paraformaldehyde (PFA) (Bio-Optica, cat: 05-K01022) and stained for ALP activity (Sigma-Aldrich Kit cat: n86C-1KT), according to the manufacturer’s instructions.

### Matrix mineralisation assay

Primary osteoblasts were cultured in DMEM with 50 μg/ml L-ascorbic acid (Sigma-Aldrich, cat: 1000528199) and 10 mM β-glycerophosphate (Sigma-Aldrich, cat: G-8501). The medium was changed every 5 days for 20–21 days. Mineralisation was detected by the von Kossa staining.

### Fluorescent phalloidin staining

Cells were fixed with 4% PFA (Bio-Optica, cat: 05-K01022) and permeabilised with Triton-X100 (Sigma-Aldrich, cat: 34H1286). Fluorescent phalloidin staining was performed according to the manufacturer’s instructions (ThermoFisher, cat: P5282).

### In vivo cancer model

To develop the in vivo cancer model, we employed the highly osteotropic and widely used human MDA-MB-231^23,24^ [MDA-MB-231 and MDA-MB-231-turboGFP (MDA^GFP^) or MDA-MB-231-Luciferase (MDA^LUC^) transfected cells] and mouse 4T1^24^ breast cancer cell line. Cells were injected into the left tibia of 5-week-old, female CD1 *nu/nu* (for human cells) or Balb/c (for mouse cells) mice (1 × 10^5^/0.01 ml PBS) and anaesthetised with intraperitoneal injection of 80 mg/kg of ketamine and 10 mg/kg of xylazine, using standard procedures.^25^ Animals were monitored daily for body weight, behaviour, and survival. Weekly, mice were also subjected to deep anaesthesia and X-ray analysis (peak kilovoltage [kVp] = 36 kV for 10 s) using a Cabinet X-ray system (Faxitron model no. 43855A; Faxitron X-Ray Corp., Buffalo Grove, IL, USA) to follow the onset and progression of osteolytic lesions. Bioluminescence evaluation was performed with the Aequoria Hamamatsu system C4742-98 to monitor tumour growth. At the end of the experiment, mice were euthanised and subjected to final X-ray analysis and anatomical dissection for evaluation of bone and visceral metastases, respectively.

### In vivo competition assay

Five-week-old female immunocompromised CD1 *nu/nu* mice or immunocompetent Balb-c mice were sub-lethally myeloablated with 100 mg/kg cyclophosphamide and 35 mg/kg bisulfan for 3 consecutive days. After further 3 days, a constant number of HSCs (50,000 cells/mouse) labelled with the red PKH26 (HSC^redPKH26^) were co-injected into the tibia medullary cavity along with increasing numbers of either MDA^GFP^ or 4T1^greenPKH67^ cells (Sigma-Aldrich, cat: MIDI67). After 1 week, bone marrow cells were recovered, and flow cytometry was used to determine the number of HSC^redPKH26^ in the bone marrow.

### Micro-computed tomography

Images from tibias fixed in 4% PFA were acquired in a SkyScan 1174 with a resolution of 6 μm (X-ray voltage 50 kV). Skyscan Nrecon software was used to employ the Feldkamp algorithm to reconstruct the images. Three- and two-dimensional (3D and 2D, respectively) morphometric parameters were calculated for the trabecular bone. Segmentation of the bone was conducted using threshold values corresponding to bone mineral density values of 0.6 cm^3^ calcium hydroxyapatite. Marching cube-type models with a rendered surface formed the basis of the 3D parameters. 2D areas were calculated using the Pratt algorithm. Bone structural variables and nomenclature were followed as suggested by Bouxsein et al.^26^

### Histology

Bone samples were decalcified for 48 h in Osteodec (Bio-Optica, cat: 05-03005E) and embedded in paraffin using the automatic paraffin embedder (Leica, TP1020). Soft organs, such as the liver and lungs, were directly embedded in paraffin. Microtome sectioning was used to obtain tissue slices of 5-µm thickness. Cells and tissue sections were labelled with antibodies against human pan-cytokeratin AE1/AE3, N-cadherin, Notch2 and Ki-67 [Santa Cruz Biotechnology, catalogue numbers, cytokeratin (sc81714), N-cadherin (sc7939), N-cadherin (sc310209), Notch2 (sc5545), Ki-67 (sc7846); Miltenyi Biotech, Notch2-Biotin (130-096-980); Novus, N-Cadherin (48309PE)], dilution 1:100–1:200, either singularly or in combinations as double, triple or quadruple immunofluorescences or stained with haematoxylin and eosin. For both cells and tissue samples, primary antibody incubations were carried out at room temperature for 1 h, then overnight at 4 °C, followed by secondary incubations for 1 h at room temperature with the corresponding secondary antibody at dilution 1:500 [*λ* 488 nm chicken anti-mouse (Life Technologies A21200), *λ* 594 nm goat anti-rabbit (Invitrogen by ThermoFisher Scientific, A11012), *λ* 405 nm streptavidin conjugate (Life Technologies, S32351), *λ* 488 nm Goat anti-mouse (Invitrogen by ThermoFisher Scientific, A11001), *λ* 594 nm rabbit anti-goat (Life Technologies, A11080)]. Immunofluorescence quantification was done using the Fiji® by Image-J software.

### Immunohistochemistry

Mouse bone samples and human primary breast cancers and bone metastases were deparaffinised and incubated with 0.07 M citrate buffer (pH 6) for 15 min at 98 °C or processed in acid unmasking buffer for antigen retrieval, then treated with 3% H_2_O_2_ and incubated overnight at 4 °C with the anti-Notch2 antibody (Santa Cruz Biotechnology, cat: sc5545) or for 1 h at room temperature with the human pan-cytokeratin AE1/AE3 antibody (DAKO, cat: M3515). The staining signals were revealed using the Dako LSAB+ System-HRP (DAKO, cat: k0679). Slides were counterstained with Mayer’s haematoxylin (Sigma-Aldrich, cat: GHS332). Positive and negative controls were performed in parallel.

### Histomorphometry

Bone sections, obtained from the different experimental settings, were stained using either commercial or clinically validated antibodies for detecting the breast cancer cells (human pan-cytokeratin AE1/AE3 DAKO®, cat: M3515, human pan-cytokeratin AE1/AE3 Santa Cruz Biotechnology, cat: sc-81714). The area of analysis was the tibial secondary spongiosa region (4 mm^2^ in area, 50 µm away from the growth plate and 20 µm away from the endocortical surface). Three to five non-consecutive tibia sections were analysed for each mouse, and the number of breast cancer cells/tissue area and their distance from the closest endosteal surface were recorded.

Liver sections obtained from the different experimental settings were stained with haematoxylin and eosin. Three non-consecutive sections were analysed for each liver, with an area of analysis/section >1 cm^2^. Sections presenting metastases were displayed in the graphs and evaluated for metastasis number/mm^2^ and for the percentage of metastasis area over total tissue area.

### Evaluation of serum interleukin (IL)-6 concentration

Enzyme-linked immunosorbent assay (ELISA) was performed on murine blood serum to quantify the levels of IL-6. The protocol was carried out according to the instructions provided in the kit (Ray-Bio, cat:622168007).

### Statistical analyses

Results are expressed as the mean ± SD. Sample size is indicated in the figure legends. Groups’ comparisons were performed carrying out independent samples *t* tests and one-way analysis of variance when dealing with continuous parameters. To avoid issues with Gaussian as well as homoscedasticity assumptions, small sample size comparisons were performed by Mann–Whitney and Kruskal–Wallis tests. First type error was set at 5%. An important need of our study was to assess the emergence of a distributional pattern of the cancer cells in the bone marrow, i.e. the lack of positional randomness. We modelled a random spot distribution of the bone cell distances, choosing a uniform distribution. A comparison between the random distances generated and the experimental distances was performed using Kolmogorov–Smirnov test. Cumulative frequency distributions were analysed also using Kolmogorov–Smirnov test. The statistical methods are indicated in the figure legends and the *p* values are indicated in the figures.

## Results

### Isolated breast cancer cells and the endosteal neighbourhood

**Fig. 1:**
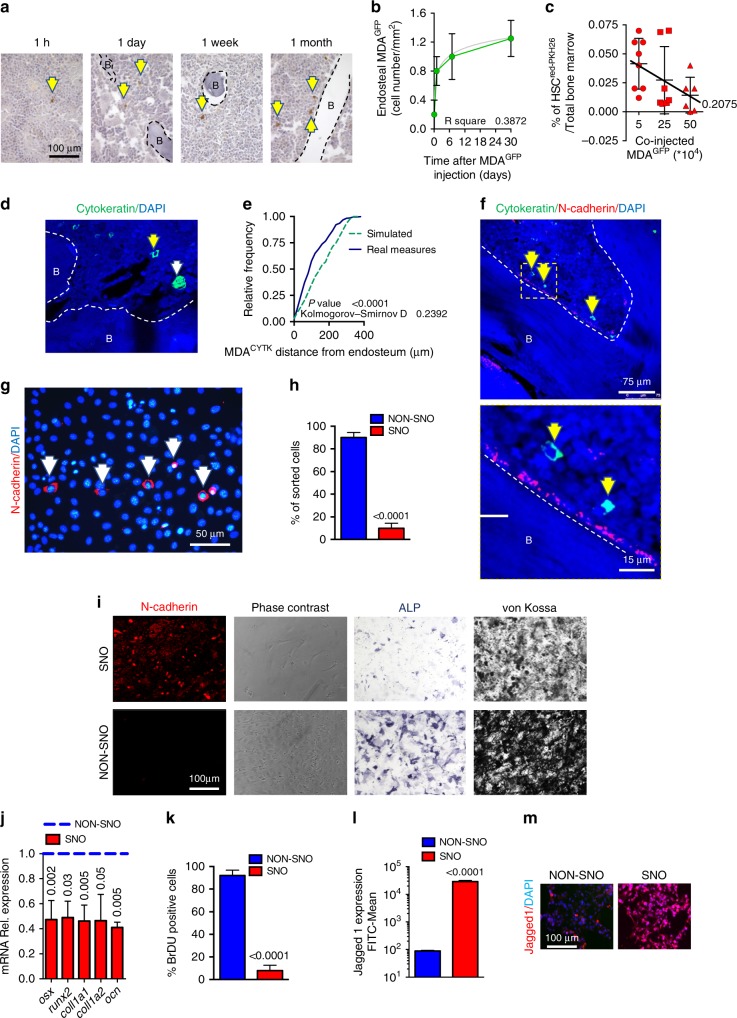
Breast cancer cells compete with haematopoietic stem cells (HSCs) for the spindle-shaped N-cadherin^+^/CD45^−^ osteoblast (SNO) endosteal niche. **a** CD1 *nu/nu* mice received an intratibial injection of MDA-MB-231-turboGFP (MDA^GFP^) cells and were randomly divided into four groups that were sacrificed at the indicated times from cell injection. The number of endosteal breast cancer cells was evaluated in non-consecutive tibia sections and results were normalised by tissue area. Representative images and **b** quantification of the number of endosteal breast cancer cells/tissue surface. B bone. **c** Sub-lethally myeloablated CD1 *nu/nu* mice received an intratibial co-injection of HSCs^red PKH26^ cells and increasing numbers of MDA^GFP^ cells. Marrow was then flushed out from the bone and subjected to flow cytometric analysis to retrieve HSC^redPKH26^ cells. **d** CD1 *nu/nu* mice received an intratibial injection of MDA-MB-231-Luciferase (MDA^LUC^) cells. After 1 month, mice that did not develop bioluminescence signal, reminiscent of lack of cancer development, were considered positive for dormancy. The tibias were isolated and stained for cytokeratin to detect breast cancer cells. Representative image by confocal laser-scanning microscopy. Yellow arrowhead, single cancer cell; white arrowhead, micro-metastasis. B bone. **e** Cumulative frequency distribution of MDA^LUC^ in the bone marrow (solid line) in comparison with the cumulative frequency distribution of random dots (dotted line). **f** Representative image by confocal laser-scanning microscopy (upper panel) and yellow square-marked inset (lower panel). Yellow arrowhead, single cancer cell. B bone. **g** Primary osteoblasts were isolated from mouse calvariae. Representative image by epifluorescence microscopy. White arrowhead, N-cadherin-positive SNOs. **h** Percentage of magnetic-activated cell-sorted SNOs and NON-SNOs. (**i**) SNO and NON-SNO characterisation for N-cadherin expression, cell morphology, alkaline phosphatase activity (4-day culture) and mineralisation ability (21-day culture in osteogenic medium; Von Kossa staining). **j** Real-time reverse transcriptase–polymerase chain reaction for the indicated osteoblast differentiation markers. **k** Bromo-deoxy-uridine (BrDU) staining. **l** Flow cytometry for the surface marker, Jagged1. **m** Jagged1 immunofluorescence. Imaging and data (mean ± SD) represent the results of at least five mice/group or three independent in vitro experiments. Statistical analyses: **b** non-linear regression analysis and curve fitting, regression curve in grey; **c** analysis of variance on trend; **e** Kolmogorov–Smirnov test; (**h**, **j**–**l**) two tails’ unpaired *t* test. DAPI, nuclear staining

Basal-like, oestrogen receptor-negative and highly osteotropic human breast cancer cell line, MDA-MB-231, stably transfected with turboGFP (MDA^GFP^), were injected into the tibia medullary cavity of 5-week-old female immunocompromised CD1 *nu/nu* mice. Groups of mice were sacrificed after 1 h to 30 days from injection, and breast cancer cell localisation was traced by immunohistochemistry for human cytokeratin. Results demonstrated that the number of isolated cytokeratin-positive breast cancer cells (MDA^CYTK^) nearby the endosteal surface increased progressively, reaching a plateau 7 days after cell injection (Fig. 1a, b).

Since the endosteal location is known to host LT-HSCs,^10,11,27^ we investigated whether these two cell types competed for bone marrow HSC engraftment in an in vivo competition assay. Therefore, a constant number of mouse HSCs (50,000 cells/mouse tibia) labelled with red PKH26 (HSC^redPKH26^) were co-injected with increasing numbers of MDA^GFP^ cells into the tibia medullary cavity of 5-week-old female immunocompromised CD1 *nu/nu* mice sub-lethally myeloablated with low doses of cyclophosphamide (100 mg/kg) and busulfan (35 mg/kg) for 3 consecutive days. After 1 week from the injection, which is the time when single tumour cell localisation adjacent to the endosteal surface reached the plateau (Fig. 1b), bone marrow cells were recovered, and flow cytometry was used to determine the number of HSC^redPKH26^ in the bone marrow. Results revealed a statistically significant inverse correlation between the number of breast cancer cells co-injected and the number of engrafted HSCs (Fig. 1c). Similar result was obtained using mouse HSC^redPKH26^ and the mouse luminal-like, oestrogen receptor-negative, breast cancer cell line 4T1^greenPKH26^ (Supplementary Fig. 1a).

### In vivo model of breast cancer dormancy

To establish an in vivo model mimicking breast cancer dormancy in the bone, mice were injected with MDA-MB-231 cells, transfected with the reporter gene, luciferase (MDA^LUC^), into the tibia medullary cavity of 5-week-old female immunocompromised CD1 *nu/nu* mice. Mice were monitored for the development of osteolytic lesions by X-ray imaging and for tumour growth by bioluminescence. Those that did not show overt signs of disease after a timeframe of 30 days were assumed to be positive for dormancy (Supplementary Fig. 1b, c). In agreement with our hypothesis, in tibias dissected from these mice, immunofluorescence for human cytokeratin revealed single MDA^CYTK^ localised adjacent to the bone surface (Fig. 1d). For statistical purpose, mathematical modelling was used to create a virtual random spot disposition inside the bone marrow and the distances of single MDA^CYTK^ from the endosteal surface were compared with the distances of 300 random spots. Cumulative frequency distribution analysis and Kolmogorov–Smirnov test demonstrated that the distribution of single breast cancer cells in the bone marrow was not random and that the area nearby the bone endosteum was their preferred site (Fig. 1e). Given that only isolated, outwardly non-proliferating tumour cells were lodged near the endosteum after 30 days from cell injection in mice not bearing overt tumours, we considered this a reliable in vivo model of breast cancer cellular dormancy.

### Endosteal niche and SNOs

Interestingly, the endosteal regions of the “dormancy mouse model” selectively lodged by single MDA^CYTK^ cells were enriched in SNOs located at the bone surface (Fig. 1f). Given the role proposed to be played by SNOs in LT-HSC quiescence^9^ and the observation that only single tumour cells were lodged in their vicinity, we postulated that SNOs could contribute to breast cancer cellular dormancy.

To demonstrate our hypothesis, we first immunophenotyped the SNOs exploiting their high expression of N-cadherin. SNOs were detected in calvarial osteoblast primary cultures and represented <10% of the total population (Fig. 1g, h). MACS was then used to purify SNOs from the N-cadherin^LOW^ osteoblast population (NON-SNOs). The phenotyping of SNOs confirmed that they were N-cadherin^HIGH^ and spindle shaped (Fig. 1i). Intriguingly, they appeared to be less differentiated than NON-SNOs, with lower ALP activity and decreased mineralisation capacity (Fig. 1i). Furthermore, the mRNA expression of the osteoblast-specific markers, *Osterix*, *Runx2*, *Collagen 1a1*, *Collagen 1a2* and *Osteocalcin*, was significantly lower in SNOs vs NON-SNOs (Fig. 1j). SNOs were also less proliferating (Fig. 1k) and less metabolically active (Supplementary Fig. 1d), and the cytoskeletal organisation showed a patchy microfilament array not typical of the osteoblast lineage^28^ (Supplementary Fig. 1e). Moreover, SNOs expressed high levels of Jagged1, a ligand for the Notch receptors, known to be involved in LT-HSC quiescence (Fig. 1l, m).

### Role of SNOs in in vitro breast cancer cell proliferation

**Fig. 2:**
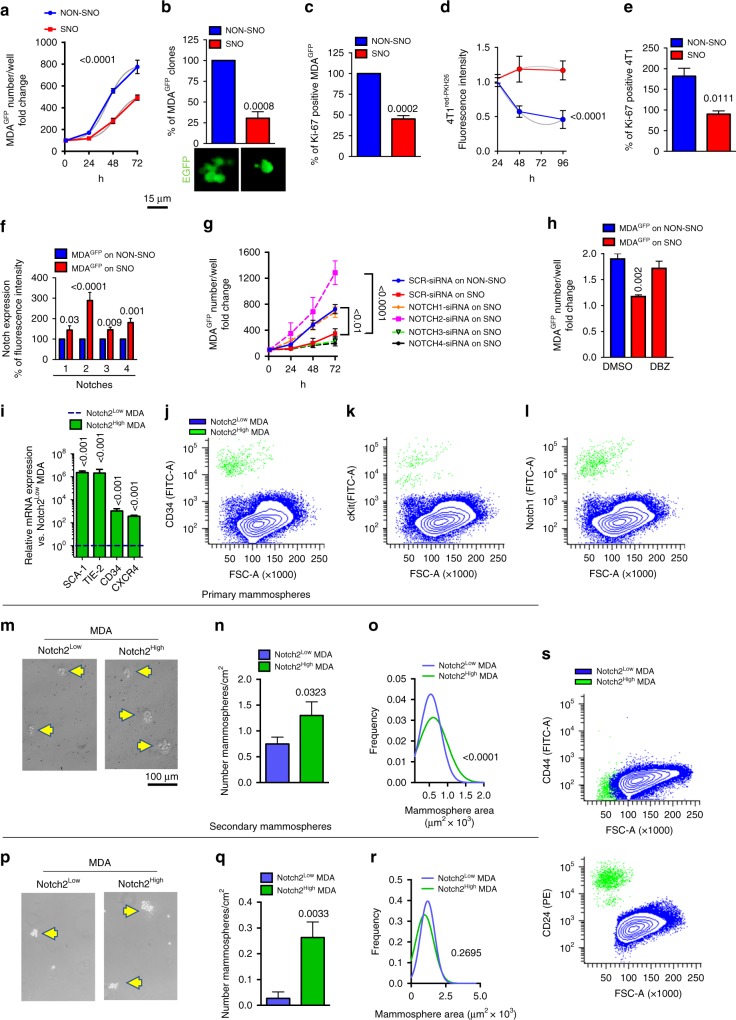
Spindle-shaped N-cadherin^+^/CD45^−^ osteoblasts (SNOs) inhibit breast cancer cell proliferation in vitro through the Notch pathway. **a** MDA^GFP^ cells were seeded onto magnetic-activated cell-sorted (MACS) SNOs and NON-SNOs cultured for 24 h and allowed to attach for 1 h at 37 °C, followed by extensive washing. Number of MDA^GFP^ cells at time = 0 (1 h of adhesion) and after 24–72 h of co-culture. **b** Number of MDA^GFP^ clones/well after 72 h of culture. **c** Number of Ki-67-positive MDA^GFP^ cells. **d** Fluorescence intensity of 4T1^redPKH26^ cells seeded onto SNO or NON-SNO monolayers after 24, 48 and 96 h of co-culture. **e** Number of Ki-67-positive 4T1 cells after 38 h of co-culture. **f** Notch1–4 expression in MDA^GFP^ cells seeded onto SNOs and NON-SNOs, evaluated after 1 h of adhesion by confocal immunofluorescence. **g** Number of MDA^GFP^ cells treated with Notch-1–4-specific small interfering RNAs (siRNAs), seeded onto SNOs and evaluated at time 0 (1 h of adhesion) and after 24–72 h, compared with SCR-siRNA-treated MDA^GFP^ cells seeded onto SNOs and NON-SNOs. **h** MDA^GFP^ cells were treated with the γ-secretase inhibitor dibenzazepine and then seeded onto SNOs. Cell number was evaluated after 1 h (time 0) and again after 24 h. The graph expresses the fold change vs time 0 and results are compared vs dimethyl sulfoxideDMSO-treated MDA^GFP^ seeded onto SNOs and NON-SNOs. **i** Transcriptional expression of the indicated haematopoietic stem cell (HSC) markers in MACS sorted Notch2^HIGH^ and Notch2^LOW^ MDA^GFP^ cell subpopulations. **j** Flow cytometry of MDA-MB-231 cells after double staining with antibodies for Notch2 and for the HSC markers, CD34, **k** c-kit and **l** Notch1. **m** Morphology, **n** number and **o** size of primary mammospheres formed by MACS-sorted Notch2^HIGH^ and Notch2^LOW^ MDA^LUC^ subpopulations. **p** Morphology, **q** number and **r** size of secondary mammospheres obtained after trypsinisation and re-plating of the primary mammospheres. Yellow arrowhead, mammosphere. **s** CD44 and CD24 expression by flow cytometry after double staining of MDA^LUC^ cells with anti-Notch2 antibody. Imaging and data (mean ± SD) represent the results of at least three independent in vitro experiments. Statistical analyses: (**a**, **d**, **g**) Non-linear regression fitting and *F*-test, (**b**, **c**, **e**, **f**, **h**, **i**, **n**, **q**) two tails’ unpaired *t* test (**o**, **r**) Gaussian curve regression fitting and *F*-test

To address the role of SNOs in tumour cell dormancy, MDA^GFP^ cells were seeded onto sorted SNOs or NON-SNOs. Although MDA^GFP^ cell adhesion measured after 1 h from plating was not statistically different in co-cultures with SNOs and NON-SNOs, during the subsequent time course of 24–72 h their number increased more slowly in MDA^GFP^-SNO than in MDA^GFP^NON-SNO co-cultures (Fig. 2a). Accordingly, MDA^GFP^ cell clones appeared to be 70% fewer in numbers after 24 h of co-cultures with SNOs (Fig. 2b). Ki-67 staining further confirmed an SNO-mediated impairment of cancer cell proliferation (Fig. 2c).

The 4T1 breast cancer cells displayed a behaviour similar to MDA^GFP^ cells. They were stained with red PKH26 and expected to lose this stable membrane dye during cytokinesis (Supplementary Fig. 2a). As anticipated, 4T1^redPKH26^ cells co-cultured with NON-SNOs showed a reduction in the red fluorescent signal that was not observed in 4T1^redPKH26^ interacting with SNOs (Fig. 2d), suggesting that 4T1 on SNOs were proliferating more slowly. The proliferation marker, Ki-67, was also lower in 4T1 cells seeded onto SNOs (Fig. 2e). Instead, no significant inhibition in proliferation was observed in the co-cultures of the luminal A-type, oestrogen receptor-positive, poorly aggressive human breast cancer MCF-7 cells, labelled with the vital fluorescent dye CMFDA (MCF-7^CMFDA^), with SNOs (Supplementary Fig. 2b), suggesting that SNO-induced quiescence is a selective and specific occurrence, likely associated with poor-prognosis breast cancers.

### Notch2 signalling

The high expression of Jagged1 in SNOs prompted us to investigate the relevance of the Jagged1 partner proteins, Notch, in the mechanism of breast cancer cellular dormancy. Single-cell analysis using confocal imaging was performed for all the Notch proteins and revealed that they were more highly expressed on the surface of MDA^GFP^ cells bound to SNOs, with the strongest expression observed for Notch2 (Fig. 2f, Supplementary Fig. 2c). The same analysis, performed on HSCs from the total bone marrow of 4-week-old CD1 mice, confirmed the Notch1, 2 and 3 proteins to be more expressed in the HSCs vs the HSC-depleted cell population (Supplementary Fig. 2d).

To investigate the functional relevance of the Notch proteins on the SNO-mediated dormancy, we silenced their mRNAs individually in MDA-MB-231 cells (Supplementary Fig. 2e) and analysed cell proliferation after seeding on SNOs during the subsequent time course of 24–72 h (Fig. 2g). This analysis demonstrated that silencing Notch2 and Notch1 reversed the SNO-mediated inhibitory effect on proliferation, with the Notch2 downregulation effect resulting more potent than the downregulation of Notch1. Accordingly, the pan-Notch inhibition using the γ-secretase blocker, dibenzazepine (DBZ), rescued the SNO-mediated dormancy of MDA^GFP^ cells (Fig. 2h). Interestingly, MCF-7 cells, whose proliferation was not affected by their interaction with SNOs (see Supplementary Fig. 2b), did not express Notch2 (Supplementary Fig. 2f), indirectly supporting a role of this pathway in the SNO-mediated dormancy process. These results prompted us to narrow down our investigation to the Notch2 pathway.

### HSC-like stem phenotype of Notch2^HIGH^ breast cancer cells

We first investigated whether MACS-isolated MDA^GFP^Notch2^HIGH^ cells (Supplementary Fig. 2g–i) showed stem features reminiscent of the HSC phenotype. Our analysis showed that they expressed high levels of mRNA for the HSC markers Stem Cell Antigen1 (*SCA1*), TEK Receptor Tyrosine Kinase (*TIE2*), *CD34* and C-X-C Motif Chemokine Receptor 4 (*CXCR4*) (Fig. 2i), and flow cytometry revealed that the MDA^GFP^Notch2^HIGH^ cell population was enriched in surface CD34, c-kit and Notch1 proteins (Fig. 2j–l), while the same markers were poorly expressed in the MDA^GFP^Notch2^LOW^ cell population (Fig. 2j–l). Taken together, these results suggest that dormant MDA-MB-231 cells mimic HSCs not only by using endosteal niche signals for quiescence but also maintaining a stem phenotype, possibly indispensable to initiate new tumours after reactivation. Of note, despite their lower proliferation in standard 2D cultures (Supplementary Fig. 2j, k), Notch2^HIGH^ cells kept the stem cell phenotype and were able to initiate both primary (Fig. 2m–o) and secondary (Fig. 2q, r) mammospheres. Moreover, mammospheres from the Notch2^HIGH^ cells were more numerous (Fig. 2n, q) and larger (Fig. 2m, o, p, r) than mammospheres generated by Notch2^LOW^ cells. Intriguingly, dormant MDA-MB-231 cell did not belong to the CD44^HIGH^/CD24^-/LOW^ breast cancer stem cell population (Fig. 2s).

### Phenotypic characterisation of Notch2^HIGH^ breast cancer cells

**Fig. 3:**
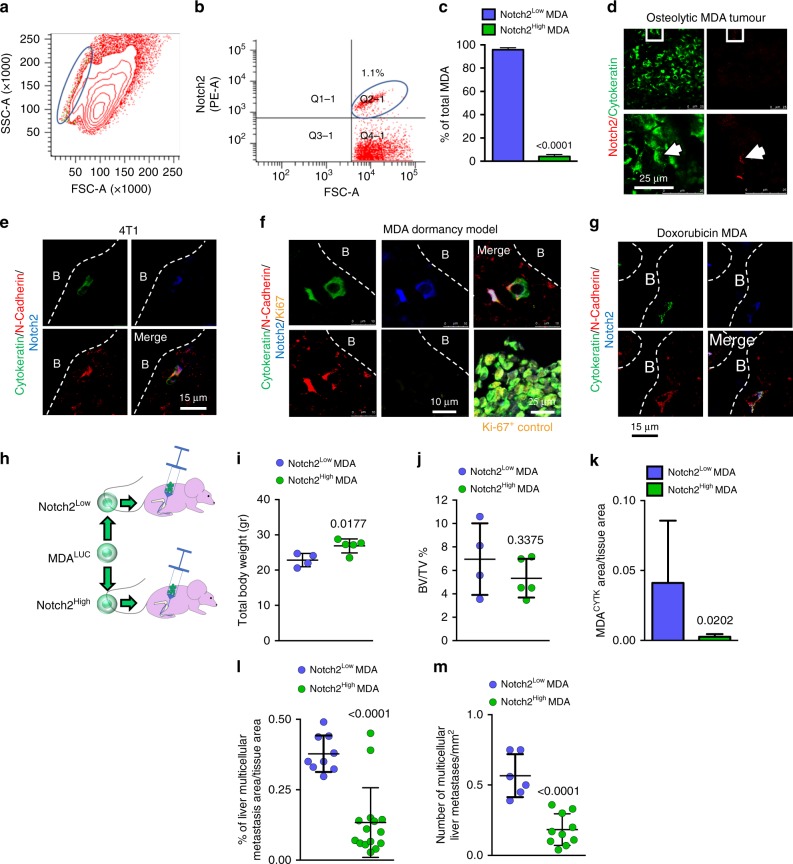
Notch2^HIGH^ breast cancer cells. **a** Size, granularity and **b** percentage of MDA^LUC^ Notch2^HIGH^ cell subpopulation after staining with anti-Notch2 antibody, analysed by flow cytometry. **c** Percentage of Notch2^HIGH^ MDA^LUC^ cell subpopulation measured after magnetic-activated cell sorting (MACS). **d** Notch2^HIGH^ MDA^LUC^ cells analysed in overt osteolytic breast cancer bone metastases. White arrowhead, Notch2^HIGH^ cell. **e** Triple immunofluorescence in the tibias of mice injected into the medullary cavity with breast cancer 4T1 cells. **f** Quadruple immunofluorescence in the tibias of the mouse model of MDA-MB-231 cell dormancy and **g** in the mouse model of chemotherapy-induced dormancy. B bone. **h** Graphical scheme of MACS-sorted Notch2^HIGH^ and Notch2^LOW^ MDA^LUC^ injection in 5-week-old female immunocompromised CD1 *nu/nu* mice. **i** Mouse body weight measured 6 weeks after cancer cell injection. **j** Micro- computed tomographic analysis of bone volume over total tissue volume (BV/TV) in tibias injected with tumour cells. **k** Immunofluorescent human cytokeratin area over tissue area, **l** percentage of metastatic area over total tissue area and **m** number of multicellular metastases per mm^2^ in the histological sections of livers collected from the mice intratibially injected with tumour cells shown in **i** and **j**. Imaging and data (mean ± SD) represent the results of at least four mice/group or three independent in vitro experiments. Statistical analyses: two tails’ unpaired *t* test

Flow cytometry performed on MDA^GFP^ cells revealed that the Notch2^HIGH^ variant belonged to a side population in the SSC/FSC plot (Fig. 3a) and represented 1–5% of the total cell population (Fig. 3b). This observation was confirmed in MDA^GFP^ cells sorted by MACS (Fig. 3c). Accordingly, immunofluorescence for Notch2 in histological sections of mouse tibias bearing MDA-MB-231 tumours revealed only a small percentage of MDA^GFP^ cells to be Notch2^HIGH^ (Fig. 3d). Similar results were obtained in the tibias of immunocompetent female Balb/c mice injected with 4T1 cells. However, owing to the potent aggressiveness of 4T1 cells, all mice developed osteolytic lesions within 15 days of cell injection (Supplementary Fig. 3a–d). Nevertheless, we observed that single cytokeratin-positive 4T1 cells lying near the N-cadherin- positive endosteal osteoblasts (Supplementary Fig. 3e) were also Notch2^HIGH^ (Fig. 3e), confirming the observations in MDA-MB-231 cells. Finally, confocal immunofluorescence against multiple antigens used to analyse tibia sections obtained from the mouse model of MDA-MB-231 cell dormancy identified MDA^CYTK^Notch2^HIGH^ cells adjacent to N-cadherin-positive osteoblasts, suggesting the in vivo reproducibility of our in vitro results. Importantly, these tumour cells were negative for the proliferation markers Ki-67 (Fig. 3f) and phospho-Histone H3b (p-HH3) (Supplementary Fig. 3f), confirming their cell cycle arrest. Of note, these tumour cells were also N-cadherin positive (Fig. 3e, f), suggesting a possible homophilic N-cadherin-mediated interactions with SNOs to occur. Finally, in vitro treatment of MDA-MB-231 cells with recombinant Jagged1-Fc, used to activate the Notch pathway (Supplementary Fig. 3g), modulated other markers of dormancy (Supplementary Fig. 3h–j), evaluated according to Johnson et al.^29^

### Resistance to chemotherapy

Since resistance to chemotherapy is a hallmark of dormant cancer cells, we tested whether single MDA-MB-231 cells were still lodging in the endosteal area after treatment with doxorubicin (Supplementary Fig. 3k). Immunocompromised female Balb/c *nu/nu* mice were subjected to intratibial injection of MDA-MB-231 cells and treated with 0.1 mg/kg doxorubicin every 2 days for 42 days. The treatment was effective in reducing the incidence (Supplementary Fig. 3l) and the size (Supplementary Fig. 3m) of osteolytic tumours in mice. Subsequently, immunofluorescence analysis revealed that in the tibia sections from the mice that did not develop tumours it was still possible to identify single MDA^CYTK^Notch2^HIGH^ cells close to N-cadherin-positive endosteal osteoblasts (Fig. 3g).

### Role of Notch2 in in vivo tumour growth

In order to investigate the specific role of Notch2 in vivo, we MACS-sorted Notch2^HIGH^ and Notch2^LOW^ MDA-MB-231 cells and injected them into the tibia medullary cavity of immunocompromised female CD1 *nu/nu* mice (Fig. 3h). After 6 weeks, mice injected with Notch2^LOW^ cells displayed a rapid and obvious weight loss (Fig. 3i), with albeit no overt sign of cachexia. μCT analysis of bone volume over tissue volume (BV/TV) showed no changes in the tibias receiving the MDA^LUC^ cells, thus ruling out the generation of osteolytic lesions (Fig. 3j). In contrast, histological analysis of bones revealed a higher number of cancer cells in Notch2^LOW^ cell-injected mice (Fig. 3k). To better investigate the cause of weight loss in MDA-Notch2^LOW^ cell-injected mice, which is normally unexpected when tumour cells are directly injected into the tibia medullary cavity, we examined anatomically the visceral organs, observing no macroscopic metastases. However, histological analysis of liver sections demonstrated that tumour cells spread to this organ forming microscopic multicellular metastases. We found that metastasis area and the number of foci were significantly higher in mice injected with Notch2^LOW^ vs Notch2^HIGH^ cells (Fig. 3l, m). We ruled out that the tumour growth in the liver was due to initial accidental seeding of tumour cells in this organ because the expression of the human *ALU* sequences after 1 h of intratibial injection was undetectable (Supplementary Fig. 3n). Altogether, these results confirmed both the relevance of the Notch2 pathway in the cellular dormancy and the role of the bone in the metastatic dissemination of breast cancers to vital visceral organs.

### Notch inhibition

**Fig. 4:**
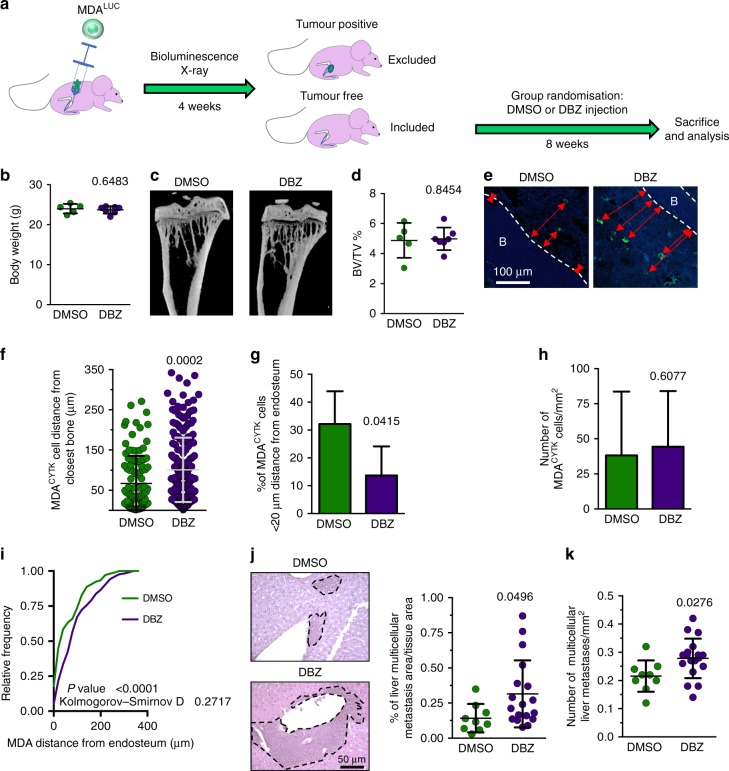
Notch inhibition. **a** Graphical representation of mice injected into the tibia medullary cavity with MDA^LUC^ cells. After 42 days from cell injection, tumour-free mice (negative to osteolysis and luciferase bioluminescence) were assumed to harbour dormant cells and were included in the study. They were randomly assorted into two groups receiving a single injection of vehicle (dimethyl sulfoxide) or of 4.28 mg/kg of the γ-secretase inhibitor dibenzazepine and sacrificed 8 weeks later. **b** Mouse body weight measured 6 weeks after cancer cell injection. **c** Micro-computed tomographic three-dimensional reconstruction of tumour cell-injected tibias. **d** Trabecular bone volume/tissue volume (BV/TV). **e** Immunofluorescence for human cytokeratin (green) in the tibias. **f** Distance of MDA^LUC^ cells from the closest endosteum. Nuclear counterstain, DAPI (blue); red arrows, distance from endosteum. B bone. **g** Percentage of cytokeratin-positive MDA^LUC^ cells located <20 µm from the closest endosteum. **h** Number of MDA^LUC^ cells/mm^2^ in the tibia. **i** Cumulative frequency distribution of MDA^LUC^ cells in the bone marrow. **j** Representative images by haematoxylin/eosin staining (left panels; dotted line, metastatic tissue), percentage of metastatic area over total tissue area (right panel) and **k** number of metastases per mm^2^ in the histological sections of livers collected from the mice displayed in **b** and **d**. Imaging and data (mean ± SD) represent the results of at least five mice/group. Statistical analyses: **i** Kolmogorov–Smirnov test; (**b**, **d**, **f–h**, **j**, **k**) two tails’ unpaired *t* test

To address the relevance of the Notch pathway in the in vivo breast cancer cellular dormancy and subsequent mobilisation, we tested whether acute Notch inhibition could reactivate the dormant breast cancer cells in our model of dormancy. After 4 weeks of in vivo dormancy (intratibial injection of MDA^LUC^ cells and absence of osteolytic lesions and bioluminescent signal), mice were randomised and divided into two groups. One group received a single acute injection of 4.28 mg/kg of the γ-secretase inhibitor DBZ,^30^ while the other group was treated with the vehicle (dimethyl sulfoxide) and used as a control (Fig. 4a). Our hypothesis was that acutely blocking the Notch signalling, by disrupting the γ-secretase activity, could destroy the interaction between SNOs and dormant breast cancer cells, mobilising and reactivating the latter. DBZ treatment was indeed effective in downregulating the Notch signalling as demonstrated by the reduced expression of the Notch downstream genes, *HRT1* and *HES1* (Supplementary Fig. 4a–c).

In the timeframe of 2 months after the DBZ treatment, we did not observe overt tumours, neither in the bone nor in the visceral organs (Supplementary Fig. 4d), and noted no significant changes in body weight (Fig. 4b). Accordingly, analysis of osteolysis using ex vivo μCT further confirmed no differences between the two groups (Fig. 4c, d). However, histomorphometric evaluation of the tibias showed that the breast cancer cell distance from the bone was significantly increased by the single DBZ treatment (Fig. 4e, f). Consequently, the number of breast cancer cells nearby the endosteal niche decreased (Fig. 4g), while the mean number of breast cancer cells per bone marrow area was not changed (Fig. 4h). Tumour cell distribution analysis confirmed that the acute Notch inhibition significantly altered the in vivo distribution of breast cancer cells in the bone marrow, inducing their mobilisation from the endosteal niche (Fig. 4i). Notably, the histological analysis of distal colonisation of the liver revealed that the number of detectable multicellular metastases, as well as their size, were increased by the acute DBZ treatment (Fig. 4j, k), suggesting mobilisation from the bone and tumour-initiating ability of reactivated cells in distant organs.

To examine whether chronic Notch inhibition could instead stimulate the local occurrence of osteolytic lesions, a similar experiment was conducted treating mice with 4.28 mg/kg DBZ 5 times/week for 5 weeks (Supplementary Fig. 4e). Even with this chronic experimental setting, no osteolytic lesions appeared in DBZ-treated mice (Supplementary Fig. 4f, g), remarking that Notch inhibition could play an important role in the induction of dormant cell mobilisation and their exit from the bone microenvironment. These results might support the concept that interference with Notch-mediated dormancy/mobilisation does not stimulate tumour growth in the bone environment but rather favours life-threatening long-distance recurrence.

### Role of inflammation

**Fig. 5:**
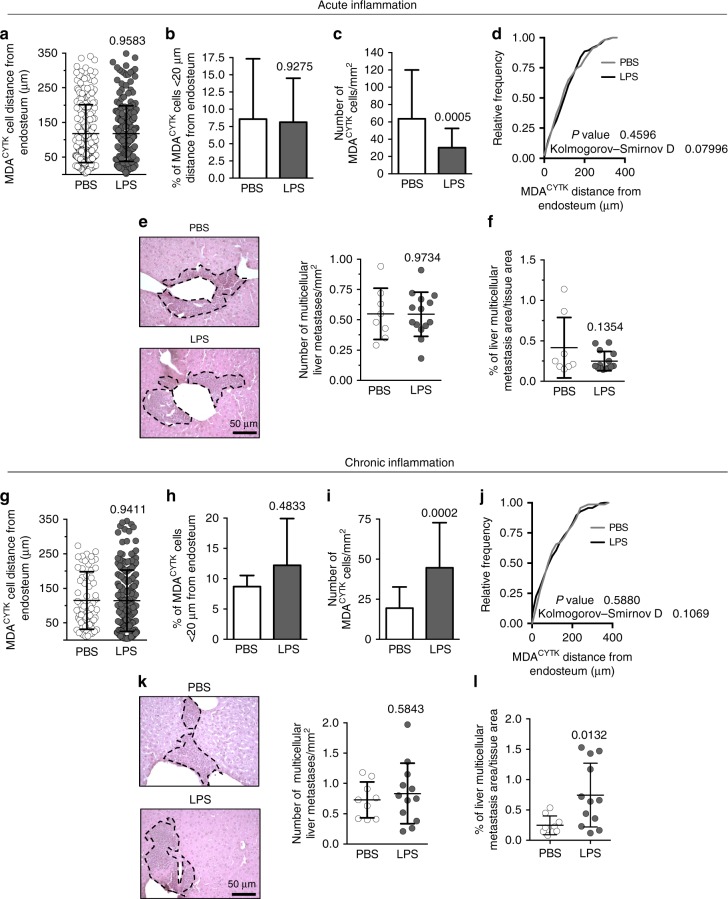
Role of inflammation. Mice with dormancy were randomly divided into two groups and treated with a single injection of 1.25 of PBS or LPS, simulating a systemic acute inflammation, and sacrificed after 8 weeks. **a** Distance of MDA^LUC^ cells from the closest endosteum. **b** Percentage of MDA^LUC^ cells located <20 µm away from the closest endosteum. **c** Number/mm^2^ of MDA^LUC^ cells in the tibias. **d** Cumulative frequency distribution of MDA^LUC^ cells in the bone marrow. **e** Representative images by haematoxylin/eosin staining (left panels; dotted line, metastatic tissue), number (right panel) and **f** the percentage of metastatic area over total tissue area in the histological sections of livers collected from 3 phosphate-buffered saline (PBS) and 5 lipopolysaccharide (LPS) treated mice. **g** Mice with dormancy were treated with daily injections of 0.25 mg/kg of PBS or LPS, simulating a systemic chronic inflammation, and sacrificed 8 weeks later to measure the distance of MDA^LUC^ cells from the closest endosteum, **h** the percentage of MDA^LUC^ cells located <20 µm away from the closest endosteum and **i** the number/mm^2^ of MDA^LUC^ cells in the tibias. **j** Cumulative frequency distribution of MDA^LUC^ cells in the bone marrow. **k** Representative images by haematoxylin/eosin staining (left panels; dotted line, metastatic tissue), number (right panel) and **l** the percentage of metastatic area over total tissue area in the histological sections of livers collected from 3 PBS and 4 LPS treated mice. Data represents the mean ± SD. Statistical analyses: **d**, **j** Kolmogorov–Smirnov test; **a**–**c**, **e**–**i**, **k**, **l** two tails’ unpaired *t* test

With the aim to broaden our findings and identify common pathological conditions that could potentially reactivate dormant breast cancer cells, we investigated the effects on dormancy of acute and chronic inflammation. After 4 weeks of dormancy (intratibial injection of MDA^LUC^ cells and absence of osteolysis and bioluminescence signal), we treated mice with a single injection of vehicle (PBS) or 1.25 mg/kg lipopolysaccharide (LPS), shown to induce an acute systemic inflammatory response (Supplementary Fig. 5a–f). In the timeframe of 2 months after the LPS treatment (Supplementary Fig. 5g), we did not observe overt tumours, neither in the bone nor in the visceral organs. Accordingly, a fine analysis of osteolysis using ex vivo μCT further confirmed no differences between the two groups (Supplementary Fig. 5h). In this model, histomorphometric evaluation of MDA^CYTK^ distance from the bone did not show significant differences between the two treatments (Fig. 5a). Similarly, the number of MDA^CYTK^ in the endosteal niche remained unchanged (Fig. 5b), although we found a decreased mean number of MDA^CYTK^ per total bone marrow area (Fig. 5c). Tumour cell distribution analysis revealed that the acute inflammation failed to alter the in vivo territorial lodging of MDA^CYTK^ in the bone marrow (Fig. 5d). Analysis of distal colonisation revealed that the number of liver metastases, as well as their size, was not changed by the treatment (Fig. 5e, f).

Subsequently, we tested whether chronic pro-inflammatory treatment could reactivate cells after 4 weeks of dormancy. Mice with breast cancer dormancy were randomised and divided into two groups, each receiving a daily injection of vehicle (PBS) or 0.25 mg/kg of LPS (Supplementary Fig. 5i). In the timeframe of 2 months of chronic LPS treatment, we did not observe overt tumours, neither in the bone nor in the visceral organs. In this model, histomorphometric evaluation of MDA^CYTK^ distance from the bone also did not show significant differences between the two treatments (Fig. 5g). Similarly, the number of MDA^CYTK^ near the endosteum was not changed (Fig. 5h). However, we found an increased number of total MDA^CYTK^ per bone marrow area (Fig. 5i) in agreement with the pro-tumour effect of chronic inflammatory stimuli. Tumour cell distribution analysis revealed that the chronic inflammation also did not alter the in vivo distribution of MDA^CYTK^ in the bone marrow (Fig. 5j). Distal colonisation revealed that the number of liver metastases was not changed by the treatment (Fig. 5k), but their size was increased (Fig. 5l).

Taken together, these results ruled out any effect of acute or chronic inflammation in the mobilisation of dormant breast cancer cells and initiation of new cancers, albeit confirming the known pro-tumour role of chronic inflammation.

### Notch2 expression in human primary tumours and correlation with survival

**Fig. 6:**
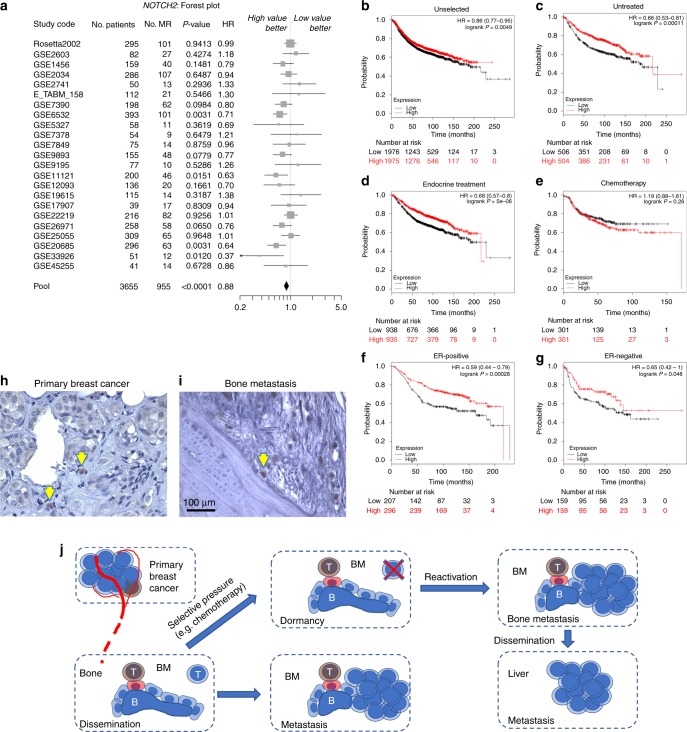
Notch2 expression in human primary tumours and correlation with survival. **a** Analysis of 23 data sets representing transcriptomes from primary breast cancers from 3655 patients and forest plot showing correlation between Notch2 and survival (SurvExpress®). **b** Kaplan–Meier plots on additional 3951 public transcriptomes from primary breast cancers to correlate Notch2 expression with patient survival in unselected populations of 1976 Notch2^LOW^ and 1975 Notch2^HIGH^ samples, **c** in untreated populations of 506 Notch2^LOW^ and 504 Notch2^HIGH^ samples, **d** in endocrine-treated populations of 938 Notch2^LOW^ and 935 Notch2^HIGH^ samples, **e** in chemotherapy-treated populations of 301 Notch2^LOW^ and 301 Notch2^HIGH^ samples, **f** in oestrogen receptor (ER)-positive of 207 Notch2^LOW^ and 296 Notch2^HIGH^ samples and **g** ER-negative populations of 159 Notch2^LOW^ and 100 Notch2^HIGH^ samples, plotted against time, (KMPlot®). **h** Primary breast cancer histopathologic samples and associated bone metastases were collected and analysed in five unselected patients. Representative immunohistochemistry for Notch2 in mated primary tumours and **i** associated bone metastasis. B bone. **j** Graphical representation summarising the findings presented in this article and their interpretation. Statistical analyses: **a** *Z*-test, **b**–**g** log-rank test

Using the SurvExpress software,^31^ we enquired 23 data sets representing breast cancers from 3655 patients. Total population analysis showed a positive statistical correlation between high Notch2 levels and overall survival (Fig. 6a). The KMplot® software^32^ microarray data of another cohort of 1809 patients were also used to test the correlation between Notch2 and survival, which appeared to be positive when we analysed the full data set (Fig. 6b), thus confirming the results obtained using the SurvExpress data set. Moreover, when we stratified patients according to the treatment, we found a positive correlation of Notch2^HIGH^ expression and survival in untreated patients (Fig. 6c) and in patients receiving endocrine treatments (Fig. 6d). Intriguingly, Notch2^HIGH^ was no longer correlated with a better prognosis under the selective pressure of chemotherapy (Fig. 6e), in line with our in vivo observation that dormant Notch2^HIGH^ breast cancer cells are resistant to doxorubicin. However, it is important to note that the divergence of the curves appears in the first 5 years, therefore we cannot exclude that they were not also due to a different rate of tumour proliferation. Furthermore, when data were stratified for oestrogen receptor subtypes, we confirmed a significant and positive correlation between high Notch2 levels and overall survival in untreated patients with either oestrogen receptor-positive or -negative breast cancers (Fig. 6f, g).

Finally, we investigated the expression of Notch2 in samples from a cohort of bone metastatic breast cancer patients both in primary tumours and matched bone metastases. We observed that in primary breast cancers Notch2^HIGH^ cells represented a small subpopulation (Fig. 6h) and that in human bone metastases they often lodged in the endosteal neighbourhood (Fig. 6i and Supplementary Fig. 6).

## Discussion

This study provides a new understanding of breast cancer cellular dormancy and mobilisation processes in the bone microenvironment. Dormant tumour cells compete with HSCs for bone marrow engraftment and lodge close to the endosteal niche made up by N-cadherin-positive osteoblasts, also known as SNOs.^9^ In this neighbourhood, they stay adjacent to the surface N-cadherin-expressing SNOs, likely by a homophilic interaction with their own N-cadherin and remain cell cycle arrested thanks to the Notch2 pathway. Disruption of this pathway reactivates dormant cells and appears to mobilise them, making their distance from SNOs significantly higher and inducing greater numbers of metastatic foci in the liver, likely through bone marrow tumour cell escaping into the circulation. In the timeframe of our experiments, such metastatic spreading induced a liver disease detectable only histologically, associated with 15% reduced body weight but not with overt cachexia, only in mice injected with Notch2^LOW^ cells, while mice treated with DBZ to inactivate the Notch signals showed normal body weight. One limitation of the study was that we were unable to demonstrate whether the lower body weight was caused by the metastatic spreading of Notch2^LOW^ cells, leaving this question unanswered.

Intriguingly, the role of the Notch pathway in tumour biology is very complex. Data in literature demonstrated that Notch1 and Notch3 are pro-oncogenic,^33–35^ and recently it has been shown that gain of Notch2 copy number is associated with poor prognosis in metastatic triple-negative breast cancer.^36^ In contrast, O’Neill et al.^37^ provided evidence that Notch2 has a marked antitumour effect in MDA-MB-231 cells, which is in line with our observations. Furthermore, Notch and its receptors have been shown to be involved in heterotypic interactions triggering escape from tumour dormancy in a model of angiogenesis-dependent cancer dormancy.^19,38–40^ While these studies clearly indicate that Notch is a key regulator of tumour dormancy, they also suggest that the outcome of Notch activation could diverge in a context-dependent manner, ranging from positive to negative. This leads to the conclusion that Notch has multifaceted roles in tumour biology and that the positive or negative outcomes are likely to depend on a fine tuning of their balance.

According to our results, the endosteal niche could be considered the place where tumour cell dormancy takes place, followed by mobilisation to initiate a new tumour when the dormancy signals are disrupted. Therefore, tumour dormancy and initiation can be considered two sides of the same coin as the long-term initiation of a new tumour is the pathologic manifestation that dormancy occurred previously.^41^

SNOs are known to induce LT-HSC quiescence through the Notch/Jagged pathway,^20^ therefore our observations favour the hypothesis that dormant breast cancer cells show similarities with HSCs and share the same niche to remain cell cycle arrested. Furthermore, dormant cancer cells share with HSCs the expression of stem genes (e.g. SCA1, CD34 and c-Kit),^42^ factors involved in their endosteal niche interaction (e.g. the members of the Notch family and Tie2)^18^ and molecular mechanisms implicated in their mobilisation (e.g. CXCR4).^43^ While the interaction with SNOs seems to be essential for dormancy, the expression of stem factors could be important for initiating new cancers when dormant cells are mobilised and reactivated.^1^ A speculative view of our results could be that the occurrence of HSC mimicry of breast cancer dormant cells makes them suitable for long-term survival in the bone marrow and that the subsequent mobilisation could lead to metastatic spread. Furthermore, like HSCs, dormant cancer cells express a drug-resistant phenotype, which contributes to their survival when cancers are challenged by chemotherapy. Given the complexity of the concept, more work will be necessary to dissect the underlying mechanisms in detail.

Notch2^HIGH^ cancer cells are also observed in human breast carcinomas. They are already detectable in primary tumours, where they represent a small portion of the total cancer cell population. In bone metastasis, Notch2^HIGH^ cancer cells are located near the endosteum. In these samples, we do not have dynamic observations that can predict the occurrence of cellular dormancy, which represent another limitation of the study. However, analysis of public data sets showed a better survival in patients bearing breast cancers expressing higher levels of Notch2, which was even more prominent in untreated patients or in patients treated with endocrine therapy. In contrast, the fact that this advantage is lost in patients after chemotherapy, suggests that drug-resistance may also be a hallmark of Notch2^HIGH^ cells in human breast cancers and that this selection can make the relapse more likely if any environmental condition mobilises and reactivates the dormant cells. Our experimental setting certainly correlated the disruption of the Notch signal with dormant cell mobilisation and colonisation of the liver. Interestingly, upon acute Notch signal disruption by DBZ, cancer cells appear unable to relocate near the endosteum, suggesting a persistent molecular change, perhaps also induced by signals emanated by the microenvironmental compartment apart from the endosteal surface. In contrast, both acute and chronic inflammation appear not to play a role in dormancy. However, in our experimental conditions, chronic inflammation increased the number of cancer cells in the bone marrow and the size of liver metastases, consistent with reports demonstrating the role of inflammation in cancer progression,^44^ but this effect seems not to be exerted on the Notch2^HIGH^ cells resident near the endosteal niche.

The paucity of Notch2^HGH^ breast cancer cells observed in vitro, in vivo and in patients strengthens the notion that they can express a stem phenotype. In fact, stem cells represent a small fraction of cancer cells,^45^ consistent with the featuring of rare mitosis, which provides self-renewal ability through asymmetric division, concurrently preventing cell exhaustion.

Interestingly, our Notch2^HIGH^ dormant cells did not express the typical stem phenotype known to characterise human breast cancer-initiating cells, CD44^high^/CD24^−/low^^46^ but rather expressed stem genes typical of HSCs. It is therefore possible that the dormant cancer phenotype we described in this study is associated with a unique HSC-like stem phenotype and that this combination is especially suitable for the long-term survival in the bone marrow. It should be noted that the phenotype we described is obvious in oestrogen receptor-negative, human and mouse breast cancer cell lines, characterised by high aggressiveness and osteotropism. In contrast, we were unable to isolate Nocth2^HIGH^ cells from the luminal-type A, oestrogen receptor-positive cell line, MCF7^47^ nor did we observe specific in vitro inhibition of their proliferation by SNOs. It is important to acknowledge that, for this aspect, mouse models do not completely recapitulate the human disease, because in contrast with luminal-like cancers, basal-like breast cancers are rarely reported to give bone metastasis in patients.^48^ This inconsistency is further suggested by the observation that high Notch2 expression in human primary breast cancers was associated with improved overall survival both in oestrogen receptor-positive and -negative breast cancer patients. Therefore, more in-depth studies will be necessary to translate the results presented in this article to the human situation.

In conclusion, we suggest that dormancy and subsequent mobilisation could feature especially aggressive osteotropic breast cancers, representing, through drug resistance and quiescence, a drawback in the current therapy, which is unable to permanently eradicate dormant cells. What we could speculatively predict as future treatment developments is a targeted therapy to make cellular dormancy permanent, thus preventing mobilisation from the dormant niche and reactivation of a proliferative phenotype. Alternatively, we could wish for an innovative strategy that could mobilise and reactivate dormant cells, making them sensitive to chemotherapy. This would allow the elimination of the dormant cell reservoir with a “shock and kill” approach, like scientists are currently endeavouring to eradicate the HIV reservoir from latently infected CD4+T cells.^49^ These strategies may represent a new frontier in medicine that in the future can save the lives of many breast cancer patients expected to experience relapse over a long time.

## Supplementary information

Supplementary material

## Data Availability

All data presented within the article and its supplementary information files are available upon request from the corresponding author.

## References

[1] Páez D, Labonte MJ, Bohanes P, Zhang W, Benhanim L, Ning Y (2012). Cancer dormancy: a model of early dissemination and late cancer recurrence. Clin. Cancer Res..

[2] Karrison TG, Ferguson DJ, Meier P (1999). Dormancy of mammary carcinoma after mastectomy. J. Natl Cancer Inst..

[3] Roodman GD (2004). Mechanisms of bone metastasis. N. Engl. J. Med..

[4] Demicheli R, Abbattista A, Miceli R, Valagussa P, Bonadonna G (1996). Time distribution of the recurrence risk for breast cancer patients undergoing mastectomy: Further support about the concept of tumor dormancy. Breast Cancer Res. Treat..

[5] Capulli M, Angelucci A, Driouch K, Garcia T, Clement-Lacroix P, Martella F (2012). Increased expression of a set of genes enriched in oxygen binding function discloses a predisposition of breast cancer bone metastases to generate metastasis spread in multiple organs. J. Bone Miner. Res..

[6] Klevesath MB, Pantel K, Agbaje O, Provenzano E, Wishart GC, Gough P (2013). Patterns of metastatic spread in early breast cancer. Breast..

[7] Harries M, Taylor A, Holmberg L, Agbaje O, Garmo H, Kabilan S (2014). Incidence of bone metastases and survival after a diagnosis of bone metastases in breast cancer patients. Cancer Epidemiol..

[8] Lynch CC (2011). Matrix metalloproteinases as master regulators of the vicious cycle of bone metastasis. Bone.

[9] Zhang J, Niu C, Ye L, Huang H, He X, Tong W-G (2003). Identification of the haematopoietic stem cell niche and control of the niche size. Nature..

[10] Calvi L, Adams G, Weibrecht K, Weber J, Olson D, Knight M (2003). Osteoblastic cells regulate the haematopoietic stem cell niche. Nature..

[11] Yin T, Li L (2006). The stem cell niches in bone. J. Clin. Invest..

[12] Bromberg O, Frisch BJ, Weber JM, Porter RL, Civitelli R, Calvi LM (2012). Osteoblastic N-cadherin is not required for microenvironmental support and regulation of hematopoietic stem and progenitor cells. Blood.

[13] Orford KW, Scadden DT (2008). Deconstructing stem cell self-renewal: genetic insights into cell-cycle regulation. Nat. Rev. Genet..

[14] Visnjic D, Kalajzic Z, Rowe DW, Katavic V, Lorenzo J, Aguila HL (2004). Hematopoiesis is severely altered in mice with an induced osteoblast deficiency. Blood.

[15] Naumov GN, Townson JL, MacDonald IC, Wilson SM, Bramwell VHC, Groom AC (2003). Ineffectiveness of doxorubicin treatment on solitary dormant mammary carcinoma cells or late-developing metastases. Breast Cancer Res. Treat..

[16] Shiozawa Y, Pedersen Ea, Patel LR, Ziegler AM, Havens AM, Jung Y (2010). GAS6/AXL axis regulates prostate cancer invasion, proliferation, and survival in the bone marrow niche. Neoplasia.

[17] Shiozawa Y, Berry JE, Eber MR, Jung Y, Yumoto K, Cackowski FC (2016). The marrow niche controls the cancer stem cell phenotype of disseminated prostate cancer. Oncotarget.

[18] Arai F, Hirao A, Ohmura M, Sato H, Matsuoka S, Takubo K (2004). Tie2/angiopoietin-1 signaling regulates hematopoietic stem cell quiescence in the bone marrow niche. Cell.

[19] Zheng H, Bae Y, Kasimir-Bauer S, Tang R, Chen J, Ren G (2017). Therapeutic antibody targeting tumor- and osteoblastic niche-derived Jagged1 sensitizes bone metastasis to chemotherapy. Cancer Cell.

[20] Sandy AR, Maillard I (2009). Notch signaling in the hematopoietic system. Expert Opin. Biol. Ther..

[21] Ilagan MXG, Kopan R (2014). Notch signaling. Methods Mol. Biol..

[22] Jarriault S, Brou C, Logeat F, Schroeter EH, Kopan R, Israel A (1995). Signalling downstream of activated mammalian Notch. Nature.

[23] Sasaki A, Boyce BF, Story B, Wright KR, Chapman M, Boyce R (1995). Bisphosphonate risedronate reduces metastatic human breast cancer burden in bone in nude mice. Cancer Res..

[24] Wright LE, Ottewell PD, Rucci N, Peyruchaud O, Pagnotti GM, Chiechi A (2016). Murine models of breast cancer bone metastasis. Bonekey Rep..

[25] Capulli M, Olstad OK, Önnerfjord P, Tillgren V, Muraca M, Gautvik KM (2014). The C-terminal domain of chondroadherin: a new regulator of osteoclast motility counteracting bone loss. J. Bone Miner. Res..

[26] Bouxsein ML, Boyd SK, Christiansen BA, Guldberg RE, Jepsen KJ, Müller R (2010). Guidelines for assessment of bone microstructure in rodents using micro-computed tomography. J. Bone Miner. Res..

[27] Xie Y, Yin T, Wiegraebe W, He XC, Miller D, Stark D (2009). Detection of functional haematopoietic stem cell niche using real-time imaging. Nature..

[28] Capulli, M., Paone, R. & Rucci, N. Osteoblast and osteocyte: games without frontiers. *Arch. Biochem. Biophys*. **561**, 3–12 (2014). 10.1016/j.abb.2014.05.003 24832390

[29] Johnson RW, Finger EC, Olcina MM, Vilalta M, Aguilera T, Miao Y (2016). Induction of LIFR confers a dormancy phenotype in breast cancer cells disseminated to the bone marrow. Nat. Cell Biol..

[30] Purow B (2012). Notch inhibition as a promising new approach to cancer therapy. Adv. Exp. Med. Biol..

[31] Aguirre-Gamboa R, Gomez-Rueda H, Martnez-Ledesma E, Martnez-Torteya A, Chacolla-Huaringa R, Rodriguez-Barrientos A (2013). SurvExpress: an online biomarker validation tool and database for cancer gene expression data using survival analysis. PLoS ONE..

[32] Györffy B, Lanczky A, Eklund AC, Denkert C, Budczies J, Li Q (2010). An online survival analysis tool to rapidly assess the effect of 22,277 genes on breast cancer prognosis using microarray data of 1,809 patients. Breast Cancer Res. Treat..

[33] Sehrawat A, Sakao K, Singh SV (2014). Notch2 activation is protective against anticancer effects of zerumbone in human breast cancer cells. Breast Cancer Res. Treat..

[34] Wang, J., Wakeman, T. P., Lathia, J. D., Hjelmeland, A. B., Wang, X.-F., White, R. R. et al. Notch promotes radioresistance of glioma stem cells. *Stem Cells* **28**, 17–28 (2009). 10.1002/stem.261 PMC2825687 19921751

[35] Xu J, Song F, Jin T, Qin J, Wu J, Wang M (2016). Prognostic values of Notch receptors in breast cancer. Tumor Biol..

[36] Stover DG, Parsons HA, Ha G, Freeman SS, Barry WT, Guo H (2018). Association of cell-free DNA tumor fraction and somatic copy number alterations with survival in metastatic triple-negative breast cancer. J. Clin. Oncol..

[37] O’Neill CF, Urs S, Cinelli C, Lincoln A, Nadeau RJ, León R (2007). Notch2 signaling induces apoptosis and inhibits human MDA-MB-231 xenograft growth. Am. J. Pathol..

[38] Chakrabarti R, Celià-Terrassa T, Kumar S, Hang X, Wei Y, Choudhury A (2018). Notch ligand Dll1 mediates cross-talk between mammary stem cells and the macrophageal niche. Science.

[39] Abravanel DL, Belka GK, Pan T, Pant DK, Collins MA, Sterner CJ (2015). Notch promotes recurrence of dormant tumor cells following HER2/neu-targeted therapy. J. Clin. Invest..

[40] Indraccolo S, Minuzzo S, Masiero M, Pusceddu I, Persano L, Moserle L (2009). Cross-talk between tumor and endothelial cells involving the Notch3-Dll4 interaction marks escape from tumor dormancy. Cancer Res..

[41] Boyerinas B, Zafrir M, Yesilkanal AE, Price TT, Hyjek EM, Sipkins DA (2013). Adhesion to osteopontin in the bone marrow niche regulates lymphoblastic leukemia cell dormancy. Blood.

[42] Spangrude G, Heimfeld S, Weissman I (1988). Purification and characterization of mouse hematopoietic stem cells. Science.

[43] Nie Y, Han Y-C, Zou Y-R (2008). CXCR4 is required for the quiescence of primitive hematopoietic cells. J. Exp. Med..

[44] Cole SW (2009). Chronic inflammation and breast cancer recurrence. J. Clin. Oncol..

[45] Wicha MS, Liu S, Dontu G (2006). Cancer stem cells: an old idea--a paradigm shift. Cancer Res..

[46] Meyer MJ, Fleming JM, Ali MA, Pesesky MW, Ginsburg E, Vonderhaar BK (2009). Dynamic regulation of CD24 and the invasive, CD44posCD24neg phenotype in breast cancer cell lines. Breast Cancer Res..

[47] Villalobos M, Olea N, Brotons JA, Olea-Serrano MF, Ruiz de Almodovar JM, Pedraza V (1995). The E-screen assay: a comparison of different MCF7 cell stocks. Environ. Health Perspect..

[48] Wei S, Siegal GP (2017). Metastatic organotropism. Adv. Anat. Pathol..

[49] Schwartz Christian, Bouchat Sophie, Marban Céline, Gautier Virginie, Van Lint Carine, Rohr Olivier, Le Douce Valentin (2017). On the way to find a cure: Purging latent HIV-1 reservoirs. Biochemical Pharmacology.

