# Supplementary material for: Notch2 pathway mediates breast cancer cellular dormancy and mobilisation in bone and contributes to haematopoietic stem cell mimicry
Source: Br J Cancer. 2019 Jun 26;121(2):157–71. doi: 10.1038/s41416-019-0501-y (PMC6738045; doi:10.1038/s41416-019-0501-y)
Supplement: Supplementary file 1 — Supplementary material [file 41416_2019_501_MOESM1_ESM.pdf]

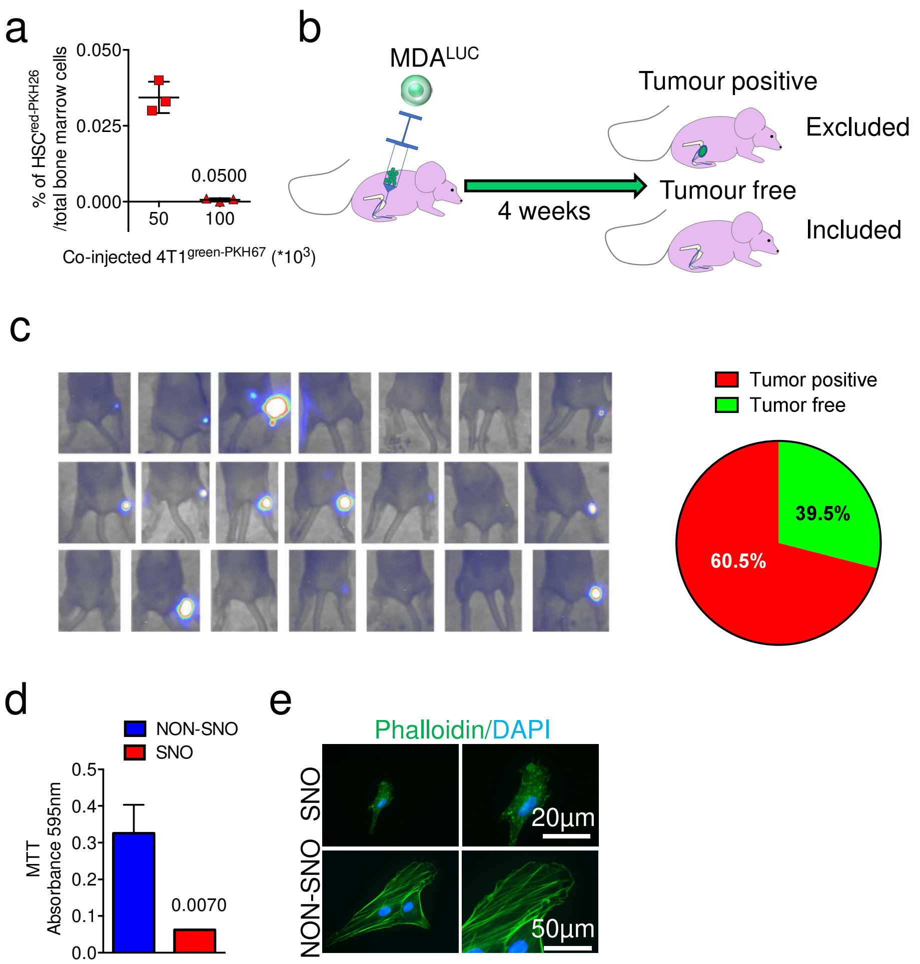


**Supplementary figure 1: HSC engraftment competition, model of dormancy and characterization of SNOs. (a)** Sub-lethally myeloablated Balb/c mice received an intratibial co-injection of a constant number HSCs$^{redPKH26}$ and increasing numbers of 4T1$^{greenPKH67}$ mouse breast cancer cells. After 1 week, the bone marrow cells were collected, and green and red fluorescent cells were measured by flow cytometry. (**b**) Graphical representation of MDA$^{LUC}$ cells intratibially injected mono-laterally in immunocompromised female CD1*nu/nu* mice. After 4 weeks mice were analysed by bioluminescence to identify overt tumours. Mice bearing tumours were excluded, while overt tumour free mice were assumed to be positive for dormancy and included in the analysis. (**c**) Representative bioluminescence images and pie-chart showing the percentage of tumour positive

mice after 4 weeks from $MDA^{LUC}$ cell injection. (**d**) SNOs and NON-SNOs were sorted by MACS, cultured for 24h and subjected to the (4,5-dimethylthiazol-2-yl)-2,5-diphenyltetrazolium bromide (MTT) reduction to formazan test for assessing cell metabolic activity. (**e**) Morphological analysis of microfilament array using phalloidin/DAPI co-staining. Imaging and data (mean$\pm$SD) represent the results 3 mice/group or 3 independent *in vitro* experiments. Statistical analyses: (a,d) two tails unpaired *t*-test.

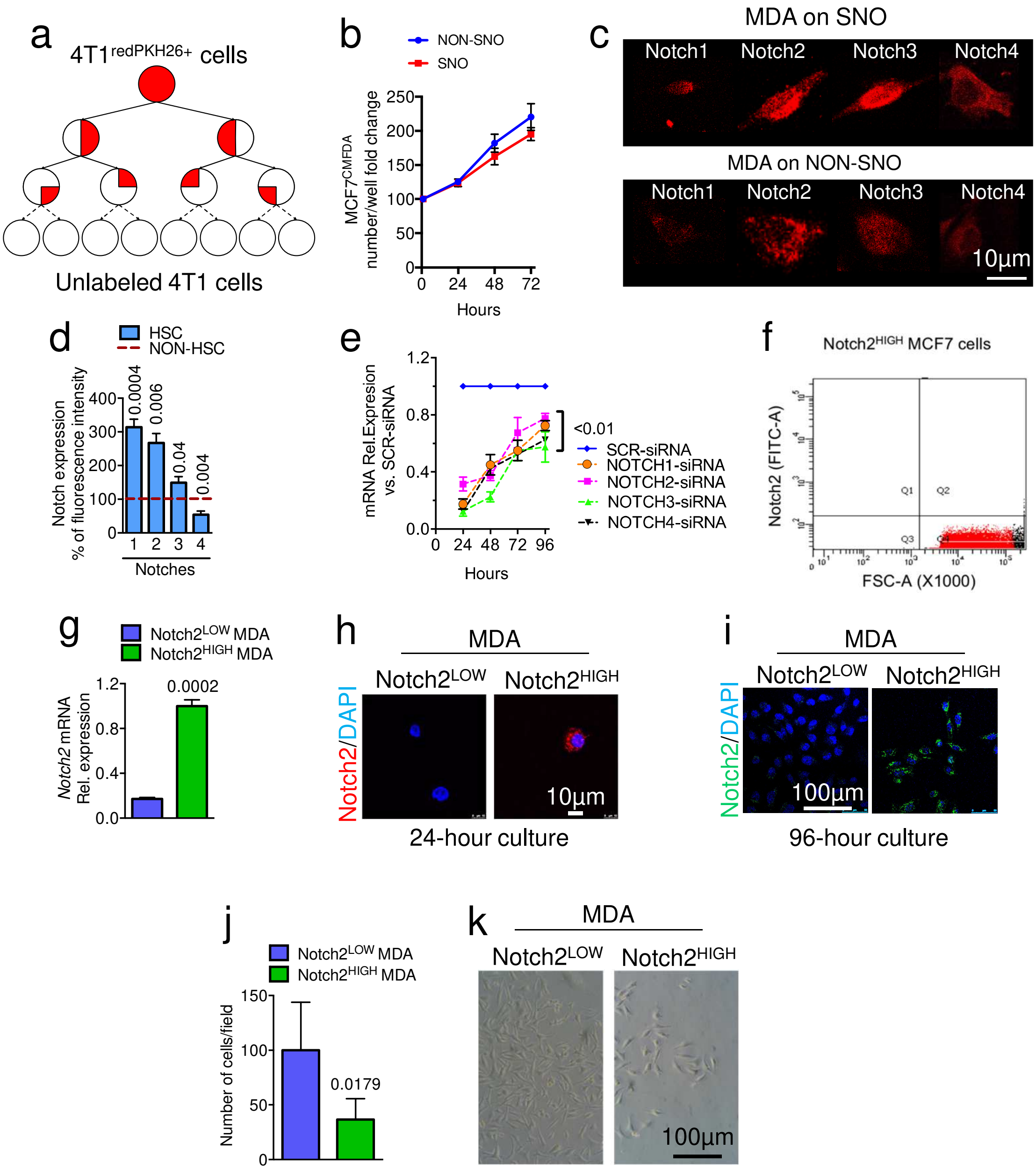


**Supplementary figure 2: Experimental strategy and Notch pathway involvement. (a)** Graphical representation of the strategy to measure 4T1 cell proliferation by fluorescent dye dilution upon cell division. (**b**) Number of human breast cancer MCF7$^{GFP}$ cells at time 0 (1h of adhesion) and after 24h to 72h of co-culture with SNOs and NON-SNOs. (**c**) Representative images of Notch 1-4 immunofluorescence performed in MDA$^{GFP}$ cells cultured on SNOs and NON-SNOs. (**d**) HSC and NON-HSC populations sorted by MACS from the total bone marrow of 1-month-old CD1 mice and analysed for Notch1 to 4 by confocal laser-scanning microscopy. Dotted line, relative value of

fluorescence of NON-HSCs. (**e**) Notch1 to 4 expression was evaluated after 24h-72h treatment of MDA-MB-231 cells with specific siRNAs targeting Notch1 to 4. Solid line, relative expression of Notch1 to 4 in scrambled siRNA transfected MDA-MB-231 cells. (**f**) Flow cytometry analysis of Nocth2 expression in MCF-7 cells. (**g**) Real-time RT-PCR Notch2 expression in MACS-sorted Notch2$^{HIGH}$ and Notch2$^{LOW}$ MDA-MB-231 sub-populations. (**h**) Notch2 immunofluorescence in MACS sorted Notch2$^{HIGH}$ and Notch2$^{LOW}$ MDA-MB-231 cell sub-populations after 24h and (i) 96h of *in vitro* cell culture. (**j**) Number and **(k)** phase contrast imaging of MACS sorted Notch2$^{HIGH}$ and Notch2$^{LOW}$ MDA-MB-231 cell sub-populations after 96h of 2D culture. Imaging and data (mean$\pm$SD) represent 3 independent *in vitro* experiments. Statistical analyses: (b,e) Non-linear regression fitting and F-test, (d,g,j) two tails unpaired *t*-test.

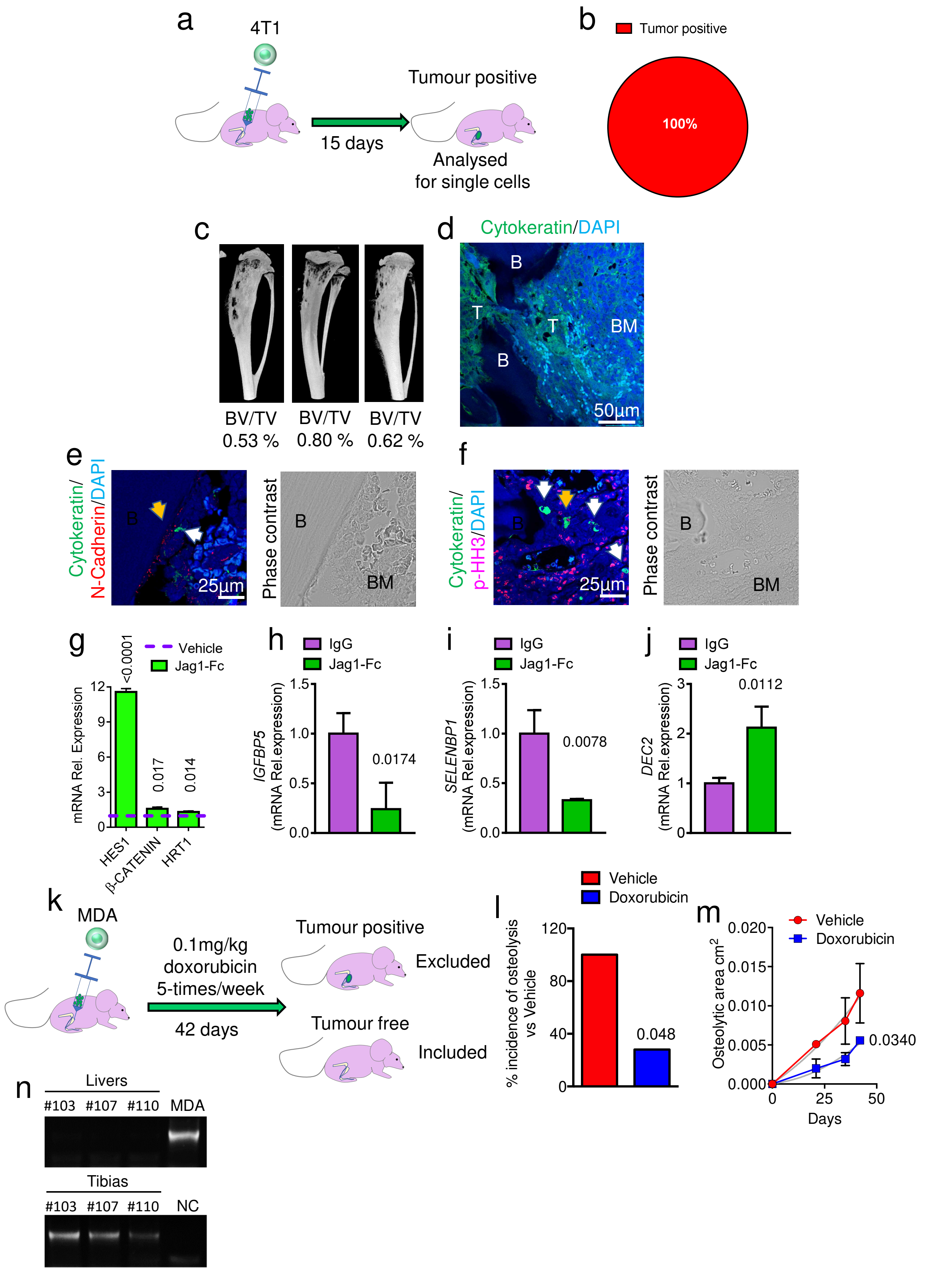


**Supplementary Figure 3: Notch2 breast cancer cells and *in vivo* experimental settings.** (a) Graphical representation of the experimental approach to obtain 4T1 osteolytic lesions in Balb/c

mice. (**b**) Pie chart showing the incidence of osteolytic lesions in Balb/c mice intratibially injected with 4T1 cells. (**c**) µCT analysis of Bone Volume/ Tissue Volume (BV/TV) in tibias collected from 4T1 cell injected Balb/c mice. (**d**) Confocal laser scanning microscopy of the tibias analysed in (c) subjected to immunofluorescence for pan-cytokeratin AE1AE3 to identify tumour cells. T, overt tumour; B, bone; BM, bone marrow; yellow arrow head, single 4T1 cells. (**e**) Fluorescence microscopy (left panel) and phase contrast imaging (right panel) of tibia section from mice injected into the medullary cavity with 4T1 cells. (**f**) Fluorescence microscopy (left panel) and phase contrast imaging (right panel) of tibia section from mice injected into the medullary cavity with MDA-MB-231 cells. (**g**) Real-time RT-PCR of the Notch2 downstream genes, *HES1, β-CATENIN* and *HRT1,* (**h**) and the dormancy associated genes *IGFBP5*, (**i**) *SELENBP1* and (**j**) *DEC2* in MDA-MB-231 cells treated with 10µg/ml recombinant Jagged1-Fc (Jag1-Fc) for 24h. (**k**) Graphical representation outlining the experimental procedures for the chemotherapy induced dormancy mouse model. Mice were treated with vehicle or doxorubicin for 42 days after intratibial tumour injection. (**l**) Incidence of osteolysis and (m) osteolytic area measured by X-ray imaging in tumour cell injected tibias. (**n**) Human *ALU* sequences evaluated by PCR in liver and tibias collected from mice 1h after the intratibial injection of MDA-MB-231 cells. Imaging and data (mean+SD) represent the results of at least 5 mice/group or 3 independent in vitro experiments. Statistical analyses: (g-j) two tails unpaired *t*-test, (l) chi-square test (m) Non-linear regression fitting and F-test. DAPI, nuclear staining.

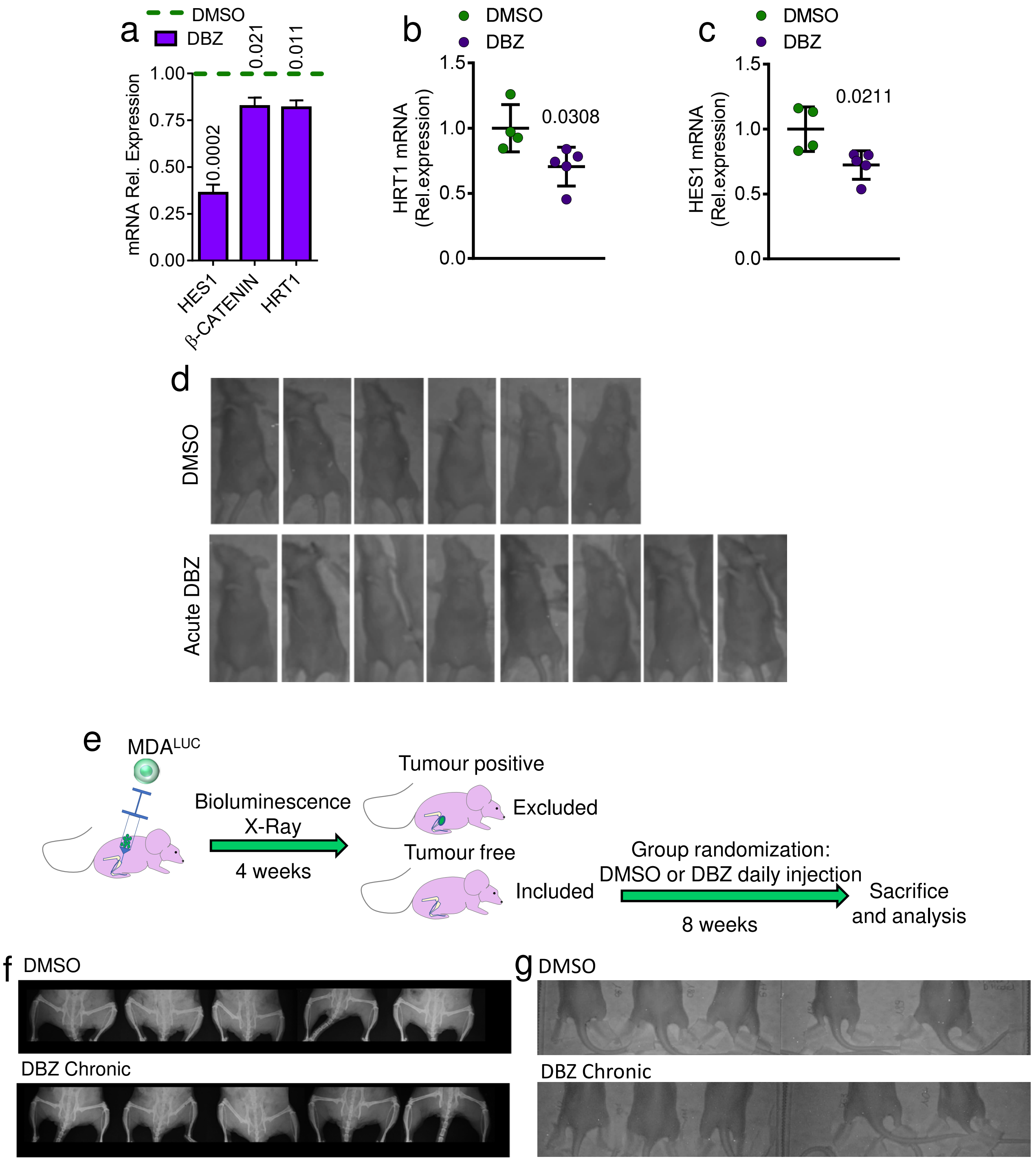


**Supplementary Figure 4: Notch inhibition.** (**a**) Representative images of mice treated as described in figure 4a. (**b**) Real-time RT-PCR of the Notch downstream genes, *HES1, β-CATENIN*, and *HRT1*, in MDA-MB-231 cells treated with vehicle (DMSO) or with 10μM of the γ-secretase inhibitor DBZ for 24h. (**c**) Real-time RT-PCR of Notch downstream genes, *HRT1* and (**d**) *HES1*, in tibias with osteolytic tumours from mice excluded from the experiment described in figure 4a,

collected after 24 hours from treatment with a single injection of vehicle (DMSO) or 4.28mg/kg of the γ-secretase inhibitor DBZ. (**e**) Graphical representation of mice injected into the tibia medullary cavity with $MDA^{LUC}$ cells. After 42 days from cell injection, tumour-free mice (negative to osteolysis and to luciferase bioluminescence) were assumed to harbour dormant cells and were included in the study. They were randomly divided into two groups receiving daily injections of vehicle (DMSO) or 4.28mg/kg of the Notch inhibitor DBZ for 6 weeks. (**f**) X-ray and (**g**) bioluminescence images of tibias injected with $MDA^{LUC}$ 6 weeks after the treatment with DBZ. Imaging and data (mean+SD) represent the results of at least 5 mice/group or 3 independent in vitro experiments. Statistical analyses: two tails unpaired *t*-test

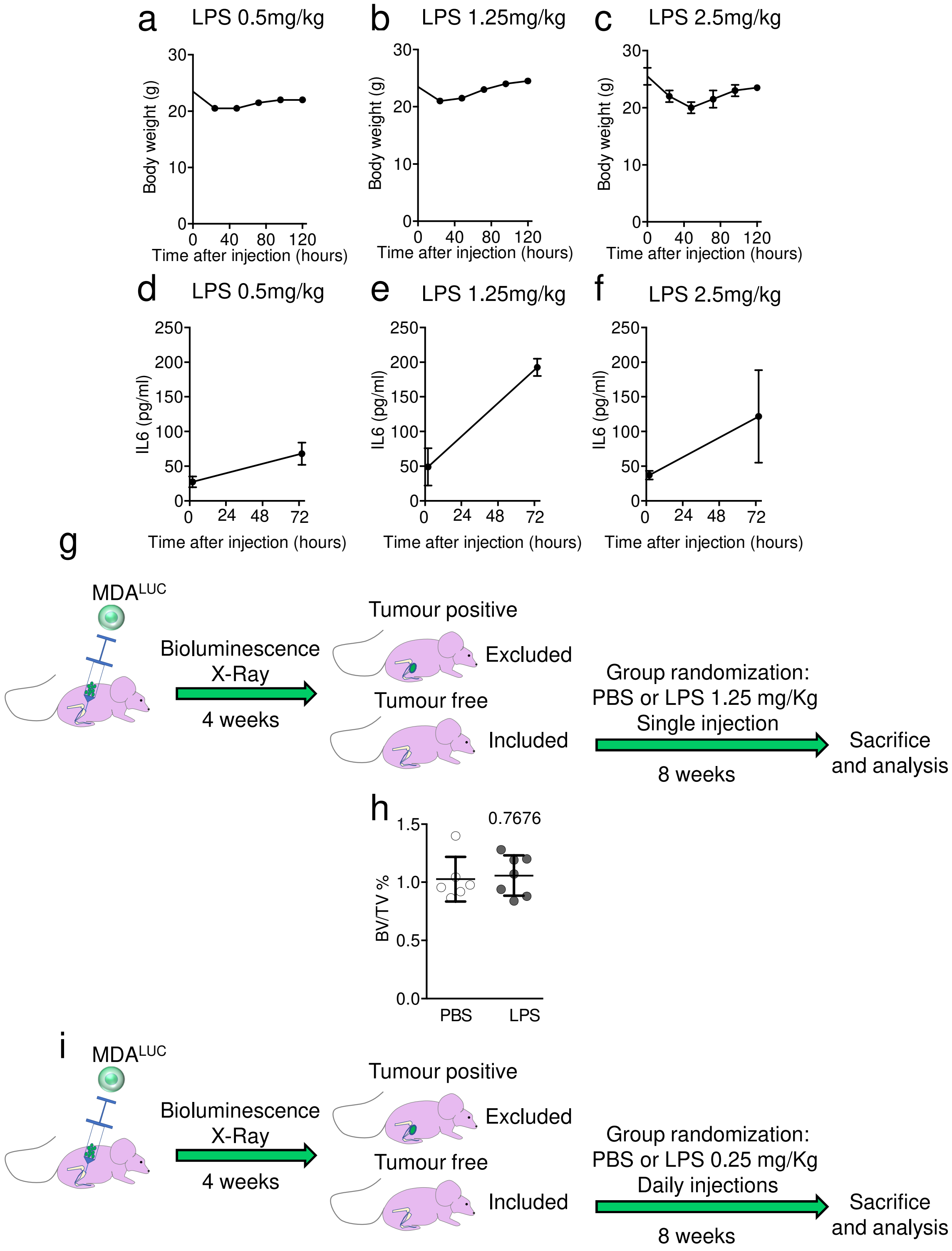


**Supplementary Figure 5: Role of inflammation.** 5-week-old CD1*nu/nu* female mice were intraperitoneally injected with (**a**) 0.5mg/kg, (**b**) 1.25mg/kg or (**c**) 2.5mg/kg LPS and body weight was monitored for 120h. (**d**) Serum concentration of the inflammatory cytokine IL-6 measured before and after 72h of treatment with 0.5mg/kg, (**e**) 1.25mg/kg and (**f**) 2.5 mg/kg of LPS. (**g**) Graphical

representation of the experimental procedures used in figures 5 (a-f). After 4 weeks of dormancy, mice were randomly divided in 2 groups and treated with a single injection of 1.25mg/kg of PBS or LPS, simulating a systemic acute inflammation. After 8 weeks from treatment, mice were sacrificed and analysed. (**h**) µCT analysis of Bone Volume/Tissue Volume (BV/TV). (**i**) Graphical representation of the experimental procedures to obtain mice used in figures 5 (g-l). After 4 weeks of dormancy, mice were randomly divided in 2 groups and treated with daily injections of 0.25mg/kg of PBS or LPS, simulating a systemic chronic inflammation. After 8 weeks from treatment, mice were sacrificed and analysed. Imaging and data (mean±SD) represent the results of at least 4 mice/group. Statistical analyses: two tails unpaired *t*-test

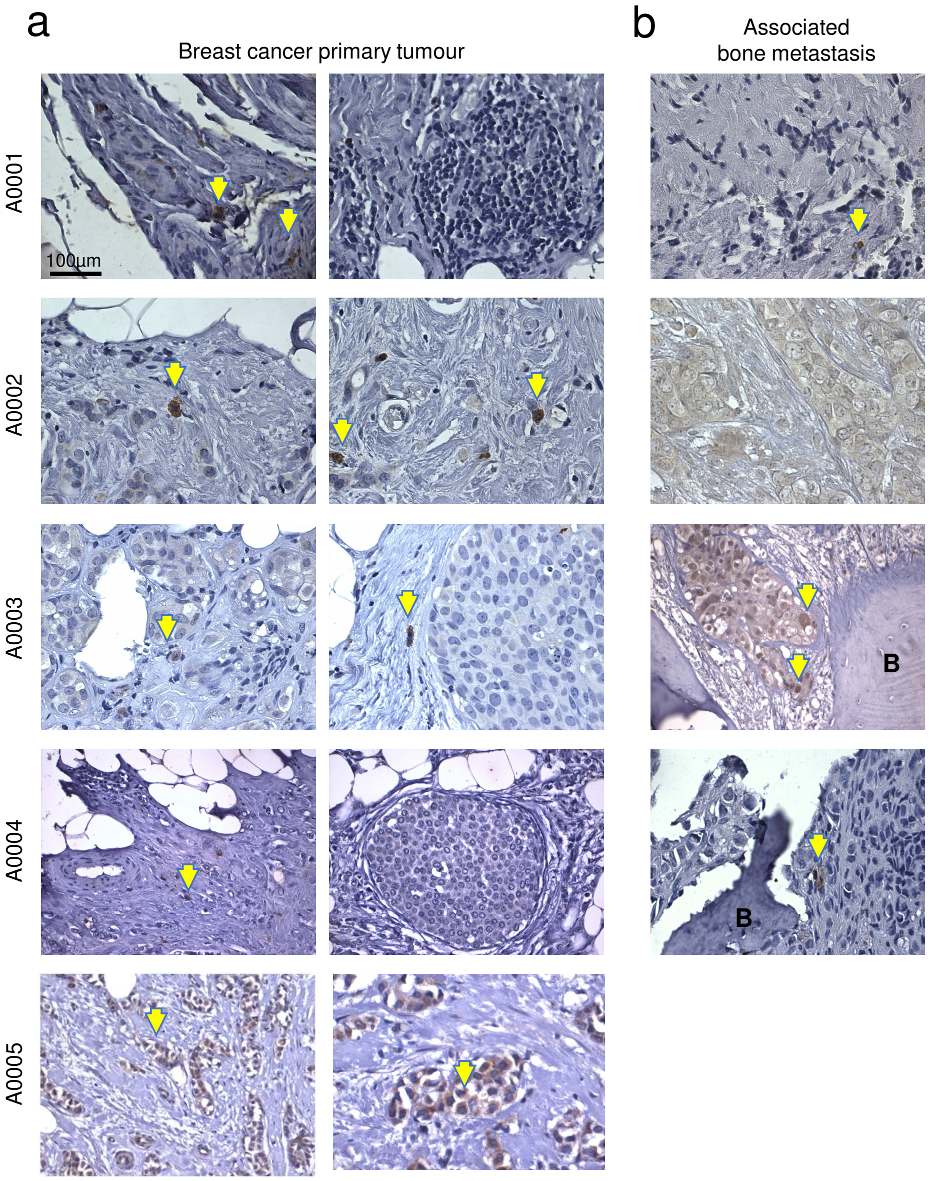


**Supplementary Figure 6. Notch2 expression in human primary breast cancers and bone metastases.** Immunohistochemistry for Notch2 in (**a**) human samples of primary breast cancer tumours and (**b**) matched bone metastases (A0001-A0005). Arrows, Notch2 positive cells; B, bone.

**Supplementary Table 1. Animal methods and ARRIVE compliance.**

| | |
|---|---|
| Ethical statement | Procedures involving animals and their care were conducted in conformity with national and international laws and policies (European Economic Community Council Directive 86/609, OJ L 358, 1, December 12, 1987; Italian Legislative Decree 116/92, *Gazzetta Ufficiale della Repubblica Italiana* no. 40, February 18, 1992; National Institutes of Health Guide for the Care and Use of Laboratory Animals, National Institutes of Health Publication no. 85-23, 1985). The procedures were approved by the Institutional Review Board of the University of L'Aquila. |
| Study design | Intratibial injection of MDA-MB-231 cells *in vivo*:<br>a. Experimental groups: 50 mice (treatment) divided after 4 weeks in two groups: tumour free (29-50%) and tumour positive (50-71%); tumour free mice further divided in 2 groups controls (single DMSO, single PBS or daily PBS injections) or treated (single DBZ, single LPS 1.25mg/kg, daily LPS 0.25 mg/kg or daily DBZ injections)<br>b. Experimental unit:<br>i. Young mice: 5-week-old female immunocompromised CD1*nu/nu* mice.<br>c. Sample that were not analysed were stored and will be used for other projects, according to the 3Rs rule (reuse)<br>Intratibial injection of MDA-MB-231 cells *in vivo* and treatment with Doxorubicin to select dormant cells:<br>a. Experimental groups: 30 mice (treatment) divided in two groups, treated daily with PBS or Doxorubicin 0.1mg/kg. After 4 weeks 80% of Doxorubicin treated mice were tumour free and used for morphological evaluations.<br>b. Experimental unit: |

| | |
|---|---|
| | i. Young mice: 5-week-old female immunocompromised CD1*nu/nu* mice.<br>c. Sample that were not analysed were stored and will be used for other projects, according to the 3Rs rule (reuse)<br>Intratibial injection of 4T1 cells *in vivo*:<br>a. Experimental groups: 10 Balb-c mice sacrificed after 2 weeks<br>b. Experimental unit: 5 weeks old, female, Balb-c immunocompetent mice<br>c. All the samples were analysed<br>*In vivo* competition assay (MDA-MB-231 and 4T1 cells):<br>a. *In vivo* competition assay: Young mice (five-week-old females) were anesthetized and injected with HSCs labelled with the stable fluorescent membrane linker, PKH26, (HSCs$^{redPKH26}$) and increasing numbers of MDA-MB-231 transfected with turbo GFP (MDA$^{GFP}$) or 4T1 cells (marked with the PKH67 green dye) into their tibia medullary cavity. These mice were also sub-lethally myeloablated.<br>b. Experimental unit: 5 weeks-old, female CD1 immunocompromised or Balb-c immunocompetent mice (for MDA$^{GFP}$ and 4T1 cell injection respectively).<br>c. All the samples were analysed |
| Experimental procedures | a. Establishing the mouse model for dormancy: mice were injected with MDA-MB-231 cells, transfected with the reporter gene, luciferase (MDA$^{LUC}$), in tibia medullary cavity of 5-week-old female immunocompromised CD1*nu/nu* mice. Mice were monitored for the development of osteolytic lesions by X-ray imaging, and for secondary tumours by bioluminescence.<br>b. X-ray imaging for osteolytic lesions: after anaesthesia, mice were exposed to X-ray imaging using the Reflotron cabinet for rodents |

| | |
|---|---|
| | c. Bioluminescence for osteolytic lesions: after anaesthesia, mice were injected with the luciferin and analysed with the Hamamatsu Aequoria system (10 minutes exposure).<br>d. Chemotherapy: Immunocompromised female Balb/c nu/nu mice were treated with 0.1mg/kg doxorubicin every 2 days for 42 days.<br>e. Injection of DBZ and DMSO: After 4 weeks of dormancy, mice were randomized and divided into 2 groups. One group received a single acute intraperitoneal injection of 4.28mg/kg of DBZ or daily injections, while the other group was treated with the vehicle (DMSO) with the same treatment regimens and used as a control.<br>f. Weighing mice: before and during the experiment<br>g. Injection of LPS and PBS: 9-weeks-old mice, after a period of 4 weeks of dormancy, received a single dose of PBS (vehicle) or 1.25mg/kg of LPS and were monitored for 8 weeks using x-ray and bioluminescent imaging. A second group of 9-weeks-old mice, after a period of 4 weeks of dormancy, were treated with daily doses of 0.25mg/kg of LPS or vehicle PBS and monitored for two months.<br>h. Sub-lethal Myeloablation of CD1*nu/nu* and Balb-c mice: young mice (five-week-old female) were sub-lethally injected with low doses cyclophosphamide and busulfan (100 mg/kg and 35 mg/kg respectively) for 3 consecutive days, and after 3 days, intratibially injected with HSC+MDA$^{GFP}$ or HSC+4T1 cells.<br>i. Anaesthesia: Ketamine/xylazine cocktail, 87.5 mg/kg Ketamine, 12.5 mg/kg Xylazine, ophthalmic ointment was applied to both eyes to prevent desiccation. To recover from anaesthesia mice were placed in warm, clean, dry, quiet environment away from other animals. Commercially-available surgical heating pad were used to warm up animals. Bedding material was |

| | |
|---|---|
| | replaced with towelling material to avoid bedding to stick to eyes or be inhaled while animals are recovering from anaesthesia.<br>j. Euthanasia: At the end of the experiments, mice (both genders) were euthanized by $CO_2$ inhalation. |
| Experimental animals | Mice used for *in vivo* studies were female CD1*nu/nu* and Balb/c strains, that were between 5-13 weeks old (according to the experimental design – figure 4a and supplementary figures 5g,i), weight 15-30 ± 3-5 gr.<br><br>Both immunocompromised and immunocompetent mice were used throughout the studies. |
| Housing and husbandry | Animal facility: standard<br><br>Temperature 20-24°C<br><br>Diet: access to food and water ad libitum, sterilized normal diet (Mucedola code: 3KE25)<br><br>Dark/light cycle: 12/12h<br><br>Humidity: 60% ± 5<br><br>Cage: plastic<br><br>Cage companions, 3 adults/cage, gender is not mixed<br><br>Bedding material: high adsorbing power, without dust. Changed every week.<br><br>Environmental enrichment was done with sterile material. |
| Sample size | 7-10 mice/group |
| Allocating animals to experimental groups | Animals were assigned to groups after randomization. |

| | |
|---|---|
| Experimental outcomes | 1. To investigate the phenotype of dormant tumour cells according to our 'mouse tumour dormancy model'<br>2. To investigate the importance of the Notch2 signalling pathway in tumour dormancy<br>3. To investigate the role played by Spindle-shaped N-cadherin$^{+}$/CD45$^{-}$ Osteoblasts in facilitating the quiescence of breast cancer cells in the bone marrow<br>4. To investigate the role of inflammation on dormant breast cancer cells |
| Statistical | Statistical analysis was performed by the Student's *t*-test and multiple comparison one-way ANalysis Of VAriance (ANOVA) according to the type of data sets. The statistical methods are indicated in the figure legends and the p-values are indicated in the figures.<br><br>Cumulative frequency distributions were tested using the Kolmogorov-Smirnoff test. The statistical methods are indicated in the figure legends and the p-values are indicated in the figures. P values of <0.05 were conventionally considered statistically significant. |

**Supplementary Table 2. List of primers**.

| Gene | Left primer | Right primer |
|---|---|---|
| | **Human** | |
| *NOTCH1* | CTTCAATGACCCCGGAAGA | GAAGTGGAAGGAGCTCTTGC |
| *NOTCH2* | ATGACTGCCCTAACCACAGG | CTGGAGTACAGGAGGCGAAG |
| *NOTCH3* | GTGTGTGTCAATGGCTGGAC | CGATAGAGCACTCGTCCACA |
| *NOTCH4* | GGCTTCTACTCCGCTTCCTT | CAACTTCTGCCTTTGGCTTC |
| *CXCR-4* | CTGAGAAGCATGACGGACAA | GACGCCAACATAGACCACCT |
| *SCA-1* | GAACCTGAAGAACGGCTCTG | GCAATCCCATTTTCTCTGGA |
| *CD34* | GCAAGCCACCAGAGCTATTC | GCCGAGTCACAATTCGGTAT |
| *TIE2* | CCAAACGTGATTGACACTGG | TGTGAAGCGTCTCACAGGTC |
| *GAPDH* | CTGCACCACCAACTGCTTA | GGTCCACCCACTGACACGTT |
| | **Mouse** | |
| *osteocalcin* | CTTGGGTTCTGACTGGGTGT | GCCCTCTGCAGGTCATAGAG |
| *osterix* | TGCTTCCCAATCCTATTTGC | AGAAATCCCTTTCCCTCTCCA |
| *runx2* | GCCGGGAATGATCAGAACTA | GGACCGTCCACTGTCACTTT |
| *collagen 1a1* | GTCCCTCTGGAAATGCTGGAC | GACCGGGAAGACCGACCA |
